# Stearoyl-CoA desaturase in CD4^+^ T cells suppresses tumor growth through activation of the CXCR3/CXCL11 axis in CD8^+^ T cells

**DOI:** 10.1186/s13578-024-01308-3

**Published:** 2024-11-14

**Authors:** Sung-Hyun Hwang, Yeseul Yang, Jae-Ha Jung, Jin Won Kim, Yongbaek Kim

**Affiliations:** 1 Laboratory of Clinical Pathology, College of Veterinary Medicine, Seoul National University, 1 Gwanak-Ro, Gwanak-Gu, Seoul, 08826 Korea; 2 BK21 Future Veterinary Medicine Leading Education and Research Center, College of Veterinary Medicine, Seoul National University, 1 Gwanak-Ro, Gwanak-Gu, Seoul, 08826 Korea; 3 Research Institute for Veterinary Science, College of Veterinary Medicine, Seoul National University, 1 Gwanak-Ro, Gwanak-Gu, Seoul, 08826 Korea; 4 Biomedical Research Institute, Seoul National University Bundang Hospital, Seongnam, 13620 Korea; 5 Department of Internal Medicine, Seoul National University Bundang Hospital, 82, Gumi-Ro 173 Beon-Gil, Bundang-Gu, Seongnam, Gyeonggi-Do 13620 Korea

**Keywords:** Tumor-infiltrating lymphocyte, CD4^+^ T cell, SCD, Lipid metabolism, CXCL11, CXCR3, CD8^+^ T cell, Immune response

## Abstract

**Background:**

Within the tumor microenvironment, altered lipid metabolism promotes cancer cell malignancy by activating oncogenic cascades; however, impact of lipid metabolism in CD4^+^ tumor-infiltrating lymphocytes (TILs) remains poorly understood. Here, we elucidated that role of stearoyl-CoA desaturase (SCD) increased by treatment with cancer-associated fibroblast (CAF) supernatant in CD4^+^ T cells on their subset differentiation and activity of CD8^+^ T cells.

**Results:**

In our study, we observed that CD4^+^ TILs had higher lipid droplet content than CD4^+^ splenic T cells. In tumor tissue, CAF-derived supernatant provided fatty acids to CD4^+^ TILs, which increased the expression of SCD and oleic acid (OA) content. Increased SCD expression by OA treatment enhanced the levels of Th1 cell markers *TBX21*, interleukin-2, and interferon-γ. However, SCD inhibition upregulated the expression of regulatory T (Treg) cell markers, *FOXP3* and transforming growth factor-β. Comparative fatty acid analysis of genetically engineered Jurkat cells revealed that OA level was significantly higher in SCD-overexpressing cells. Overexpression of SCD increased expression of Th1 cell markers, while treatment with OA enhanced the transcriptional level of *TBX21* in Jurkat cells. In contrast, palmitic acid which is higher in SCD-KO cells than other subclones enhanced the expression of Treg cell markers through upregulation of mitochondrial superoxide. Furthermore, SCD increased the secretion of the C–X–C motif chemokine ligand 11 (CXCL11) from CD4^+^ T cells. The binding of CXCL11 to CXCR3 on CD8^+^ T cells augmented their cytotoxic activity. In a mouse tumor model, the suppressive effect of CD8^+^ T cells on tumor growth was dependent on CXCR3 expression.

**Conclusion:**

These findings illustrate that SCD not only orchestrates the differentiation of T helper cells, but also promotes the antitumor activity of CD8^+^ T cells, suggesting its function in adverse tumor microenvironments.

**Graphical abstract:**

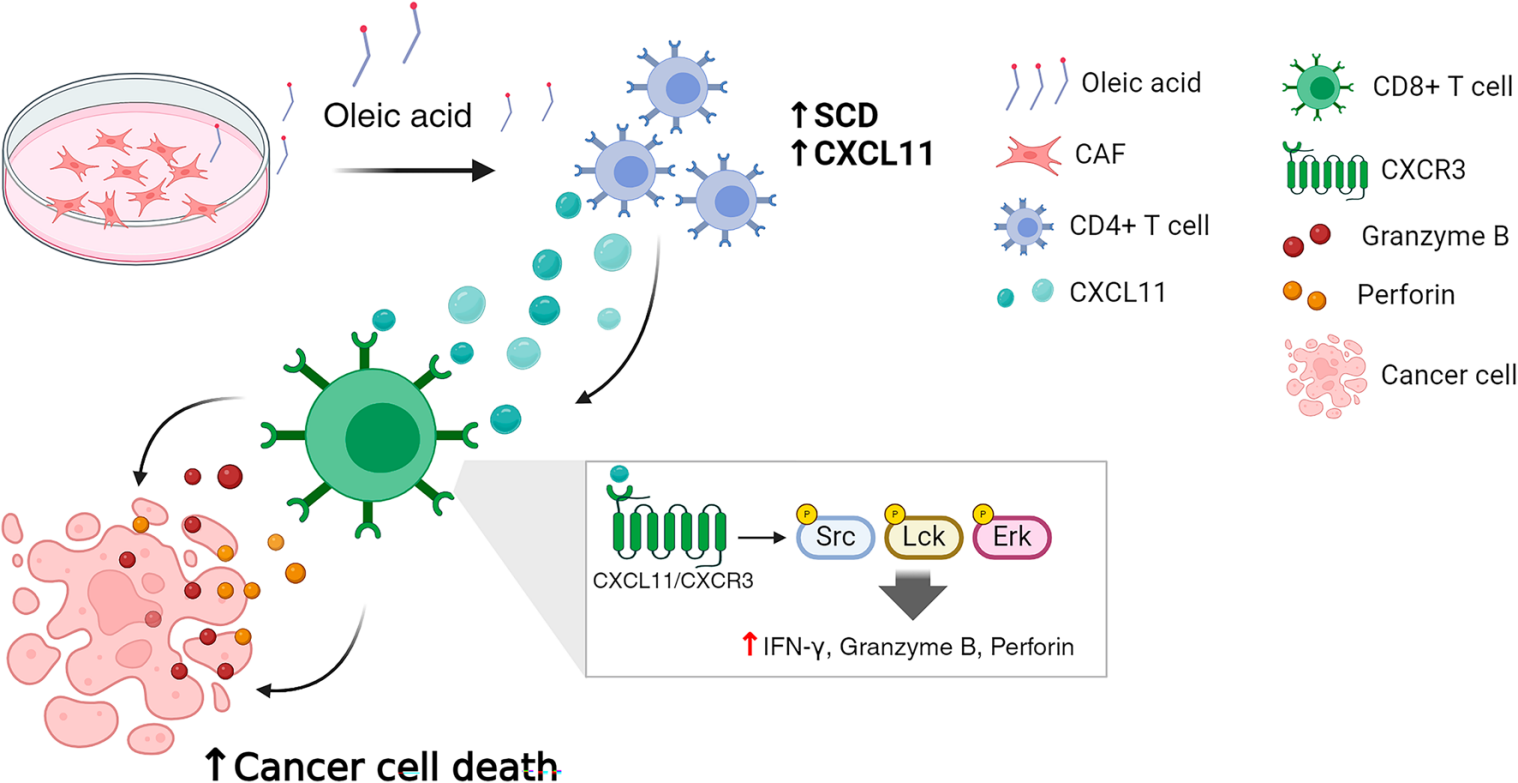

**Supplementary Information:**

The online version contains supplementary material available at 10.1186/s13578-024-01308-3.

## Background

In the tumor microenvironment, abnormal metabolic activity of cancer cells induces metabolic alterations in non-cancerous cells [1, 2]. Cancer-associated fibroblasts (CAFs) surrounding the cancer cells exhibit a reprogrammed metabolism that is different from normal fibroblast, promoting the resistance to anticancer drugs and tumor progression [3]. Additionally, the reprogrammed metabolism of CAFs activates lipid metabolism in cancer cells. Our previous study showed that fatty acids (FAs) derived from CAFs are transferred to neighboring cancer cells via supernatant fluid, resulting in the upregulation of tumor progression [4]. However, our understanding about the effects of CAF-derived lipid metabolites on tumor-infiltrating lymphocytes (TILs) is limited. In naïve CD4 T cells, translational machinery enhances metabolic reprogramming, including fatty acid synthesis, to promote differentiation into an effector phenotype [5]. Notably, enhanced lipid metabolism preferentially expands regulatory T (Treg) cells over other cell subsets, mediated by the phosphorylation of AMP-activated protein kinase [6]. Moreover, FA metabolism promotes the differentiation of naïve T cells towards interferon-γ (IFN-γ)- and CD4-positive T cells [7]. Conversely, inhibiting lipid metabolism suppresses Th1 cell function [8].

Adipose tissue is physically proximal to lymphoid tissue and alters metabolic signaling by supplying FAs, thereby promoting the self-renewal of lymphocytes [9]. FAs, produced by acetyl-CoA and NADPH in the mitochondria, cross the cell membrane via CD36. In cancer cells, FAs strengthen the maintenance of structural integrity and fluidity, and they activate oncogenic signaling, such as PI3K-AKT signaling, thereby enhancing chemoresistance [10]. Additionally, FAs promote lymphocyte proliferation, with higher levels in CD4^+^ TILs than in splenic CD4^+^ T cells [11].

FAs are classified as either saturated or unsaturated. The conversion of saturated fatty acids (SFAs) to unsaturated fatty acids (UFAs) by stearoyl-CoA desaturase (SCD) not only augments ATP synthesis in mitochondria but also suppresses apoptosis caused by peroxidation [12]. SCD activates lipid metabolism by upregulating carnitine palmitoyltransferase-1 and acyl-CoA [13]. Furthermore, the effects of FAs on T cells have been extensively studied. Treatment with palmitic acid (PA), an SFA, enhances cell death and suppresses the activity of stimulated T cells. These effects are ameliorated by treatment with oleic acid (OA), a UFA [14]. Additionally, the expression of IL-2 in CD4^+^ T cells is enhanced by OA, but not by SFA [15, 16].

The cytotoxic efficacy of CD8^+^ T cells depends on the composition of CD4^+^ T cell subsets within the tumor tissue. In a microenvironment dominated by Treg cells, cancer cells evade surveillance by cytotoxic T cells, thereby promoting tumor growth. Conversely, cytokines such as IFN-γ and IL-2, produced by Th1 cells, enhance the cytotoxic effect of CD8^+^ T cells [17, 18]. Additionally, C-X-C chemokine ligands 9, 10, and 11 (CXCL9, CXCL10, CXCL11) secreted by CD4^+^ T cells interact with C-X-C chemokine receptor 3 (CXCR3) on CD8^+^ T cells, which activates and expands these cytotoxic cells [19]. Injection of these ligands into tumor tissues recruits natural killer cells, cytotoxic lymphocytes, and M1 macrophages, thereby effectively suppressing tumor growth [20].

## Methods

### Cell lines and reagents

Mouse mammary carcinoma 4T1 cells and human T leukemia Jurkat cells were cultured in complete Roswell Park Memorial Institute (RPMI) medium (Gibco-Life Technology, Gaithersburg, MD, USA) supplemented with 10 mM glucose, 100 μg/mL penicillin/streptomycin (Gibco), 1 mM sodium pyruvate (Sigma-Aldrich, St. Louis, MO, USA), 10 mM HEPES (Sigma-Aldrich), 10 mM sodium carbonate (Sigma-Aldrich), and 10% fetal bovine serum (Gibco). Mouse colorectal cancer CT26 and melanoma B16F10 cell lines were cultured in Dulbecco’s modified Eagle’s medium (Sigma-Aldrich) supplemented with identical components to complete RPMI medium. After transfection with the Luc2-vector (Promega, Madison, WI, USA), Luc2-4T1 cells were selected using G418 (Sigma-Aldrich).

Sulfosuccinimidyl oleate (SSO; Cayman Chemical, Ann Arbor, MI, USA) and AMG487 (Cayman Chemical) were used to inhibit lipid transporters and CXCR3, respectively. CAY10566 (Cayman-Chemical) and T0901317 (Cayman Chemical) were used to inhibit and enhance the expression of SCD, respectively. OA (Sigma-Aldrich) and PA (Sigma-Aldrich) were dissolved in dimethyl sulfoxide (Sigma-Aldrich). A cell stimulation cocktail (Invitrogen, Carlsbad, CA, USA) was added for 18 h to stimulate the lymphocytes. The cells were then treated with 10 ng/mL recombinant CXCL11 (rCXCL11) (R&D Systems, Minneapolis, MN, USA) for 1 d.

### Mouse models for tumor growth assays and primary culture

All animal experiments were approved by the Institutional Animal Care and Use Committee of the Seoul National University, South Korea (SNU-200309-9). Four-week-old BALB/c mice were purchased from Jungang Lab Animal, Inc. (Seoul, South Korea) and injected with 2 × 10^5^ 4T1 cells into the mammary fat pad. The tumor volume was calculated as (width^2^ × height)/2. When the tumor size reached 100 mm^3^, the mass was subjected to primary culturing for CAFs and TILs. Splenocytes were isolated from 4-week-old female BALB/c mice according to a previously published method [21]. The process for isolation of CAFs, CD3^+^CD4^+^ cells, and CD3^+^CD8^+^ cells from primary cells are described in Sect. “Isolation of CAFs and CD3+CD4+ and CD3+CD8+ cells from primary culture”.

For the transplantation experiments, 2 × 10^5^ Luc2-4T1 cells were injected into immunocompetent BALB/c mice. When the tumor size reached 150 mm^3^, the mice received the following treatments: 1) one-time intraperitoneal injection with phosphate-buffered saline (PBS) or 2 × 10^7^ CD8^+^ T cells or CXCR3-CD8^+^ T cells and 2) intraperitoneal injection once weekly with 100 μg of anti-PD1 (RMP1-14; BioXCell, Lebanon, NH, USA). To monitor tumor growth, the bioluminescence of Luc2-4T1 cells was assessed using an IVIS Lumina XR system (Caliper-Life Sciences, Waltham, MA, USA) after administration with 150 μg of VivoGlow Luciferin (Promega). The schedule for the tumor growth model is shown in Supplementary fig. S6A and S6E. Images were analyzed using Living Image software (Caliper Life Sciences). The background bioluminescence was 2.1 × 10^7^ photons/s.

### ***Isolation of CAFs and CD3***^+^***CD4***^+^***and CD3***^+^***CD8***^+^***cells from primary culture***

CAFs and CD3^+^CD4^+^ and CD3^+^CD8^+^ cells were isolated from primary cells derived from the tumor mass and splenocytes using commercial kits (Miltenyi Biotec, Bergisch Gladbach, Germany), according to the manufacturer’s instructions. Isolated cells were cultured in complete RPMI medium. For purity analysis, the isolated cells were stained with primary antibodies including anti-fibroblast activation protein (FAP; AbCam, Cambridge, UK), anti-CD3 (Invitrogen), anti-CD4 (Invitrogen), and anti-CD8 (Invitrogen), each diluted at a 100:1 ratio in 1% bovine serum albumin (BSA) in PBS at 4 °C for 1 h. After washing with PBS, secondary antibodies that were conjugated with fluorescence were diluted (200:1) in PBS and incubated at room temperature in the dark for 1 h. The cells analyzed using a FACSVerse flow cytometer (BD Biosciences, San Jose, CA, USA).

### Establishment of genetically engineered cells

To generate SCD-knockout (KO) Jurkat cells, either the control or SCD-sgRNA-U6 plasmid (Suppl. Table S1; Toolgen, Seoul, South Korea) were transfected with the CRISPR/Cas9-puromycin-CMV plasmid vector (Toolgen) and Lipofectamine-3000 (Invitrogen). After selection in 2 μg/mL puromycin, resistant cells were individually cultured, and SCD expression was assessed by western blotting.

The amplified polymerase chain reaction (PCR) product of the *SCD* coding sequence was inserted into the TOPO vector (Invitrogen) and ligated into the pLenti6.3/V5-DEST vector (Invitrogen). The prepared SCD-pLenti and empty pLenti vectors were transfected into 293 T cells with the 2nd lentivirus packaging solution (Applied Biological Materials, Richmond, BC, Canada) and lentifectin (Applied Biological Materials), respectively. After 2 d, the supernatant containing the virus was harvested and concentrated using a Lenti-X concentrator (Takara, Otsu, Japan). Jurkat cells were infected with either control or SCD-pLenti viral supernatants using polybrene (Merck Millipore, Guyancourt, France) and selected with G418.

The CXCR3-pLenti viral supernatant was prepared using the TOPO and pLenti6.3/V5-DEST vectors, following the above protocol, and infected CD8^+^ T cells were isolated from splenocytes. After selection with G418, the expression of CXCR3 was assessed.

### Apoptosis assay

Cancer cells were co-cultured with CD8^+^ T cells at a 1:10 ratio for 24 h. After discarding the supernatant containing CD8^+^ T cells, an apoptosis assay was performed using the Ezway Annexin V Apoptosis Detection Kit (Koma Biotech, Seoul, South Korea) according to the manufacturer’s instructions [22]. The remaining CD8^+^ T cells were excluded based on differences in the sizes of the forward and side scatter.

### Measurement of lipid content and FA subtypes

Lipid concentrations in the cultured cells were measured by staining with BODIPY 493/503 (Invitrogen) according to the manufacturer’s instructions. Briefly, the harvested cells were incubated with a 10 μM BODIPY solution at 37 °C in the dark for 30 min. After washing three times with PBS, the cells were analyzed using a FACSVerse flow cytometer (BD Biosciences).

The composition of the FAs extracted from the harvested cell pellets was analyzed using fatty acid methyl esters at the National Instrumentation Center for Environmental Management at Seoul National University [23].

### Measurement of reactive oxygen species

The levels of intracellular reactive oxygen species (ROS) and mitochondrial superoxide were measured using a 2,7-dichlorodihydrogluorescein diacetate assay (DCF-DA, Invitrogen) and MitoSOX (Invitrogen), respectively. Briefly, harvested cells were stained with 2 μM DCF-DA for 30 min in the dark to assess the ROS levels. Mitochondrial superoxide levels were assessed by staining with 5 μM MitoSOX for 15 min in the dark. After washing with PBS, the fluorescence of DCF-DA-FITC and MitoSOX-PE was immediately analyzed using a FACSVerse flow cytometer (BD Biosciences).

### Expression analysis by quantitative reverse transcription-PCR and western blotting assays

Total RNA was extracted using TRIzol reagent (Ambion, Austin, TX, USA) and quantified using a microplate reader (BioTek Epoch, Izasa, Barcelona, Spain). To synthesize cDNA, 500 ng of total RNA was used with the TOPscript™ cDNA synthesis kit (Enzynomics, Daejeon, South Korea). mRNA expression was evaluated using a SYBR Green reverse transcription-PCR kit (Enzynomics). Relative expression levels were normalized to GAPDH expression levels, and were calculated according to the ΔΔCt method [24]. The primer sequences are listed in Suppl. Table S1.

Protein was extracted using EzRIPA buffer (ATTO, Tokyo, Japan) and its concentration was assessed using the Bradford assay (Bio-Rad, Hercules, CA, USA). Membranes loaded with 10 μg of protein lysate were incubated with skim milk solution (PBS with 0.1% Tween 20 [PBS-T] + 5% skim milk) for 1 h. After washing three times with PBS-T, the membranes were incubated overnight at 4 °C with a blocking solution (PBS-T + 5% bovine serum albumin) containing primary antibodies against liver X receptor (LXR; Santa Cruz Biotechnology, Dallas, TX, USA), SCD (Cell Signaling Technology, Danvers, MA, USA), sterol regulatory-element binding protein (Santa Cruz Biotechnology), and GAPDH (Santa Cruz Biotechnology), each diluted at a 1000:1 ratio in 1% BSA in PBS-T overnight at 4 °C. After washing with PBS-T, the membranes were incubated with a solution containing secondary horseradish peroxidase (HRP)-conjugated anti-rabbit and anti-mouse antibodies (Santa Cruz Biotechnology) diluted at a 2000:1 ratio in skim milk for 2 h. Protein expression was detected using a ChemiDoc Touch Imaging System (Bio-Rad, Hercules, CA, USA) after adding the Luminata Forte Western HRP Substrate (Merck Millipore).

### Detection of cytokine expression by flow cytometric analysis and enzyme-linked immunosorbent assays

To measure the intracellular protein expression levels in stimulated Jurkat cells and CD4^+^ and CD8^+^ T cells with Stimulation Cocktail (Invitrogen), the cells were fixed and permeabilized using Intracellular Fixation & Permeabilization Solution (Invitrogen), followed by incubation with antibodies against IFN-γ (Invitrogen), FoxP3 (Invitrogen), T-bet (BioLegend, San Diego, CA, USA) and CXCR3 (Invitrogen) at a 100:1 ratio for 1 h. After washing with PBS, protein expression was immediately analyzed using a FACSVerse flow cytometer (BD Biosciences).

The concentrations of transforming growth factor-β (TGF-β), interleukin-2 (IL-2), and tumor-necrosis factor-α (TNF-α) were determined using commercial enzyme-linked immunosorbent assay kits (Duoset, Minneapolis, MN, USA). Briefly, the standards and supernatants collected from stimulated Jurkat cells and CD4^+^ T cells were suspended on a plate pre-coated with each antibody and incubated for 2 h. After washing three times with PBS-T, the detection antibody and streptavidin-HRP solution were added to the plate for 2 h and 30 min, respectively. Tetramethylbenzidine (Sigma-Aldrich) was added for 20 min to monitor the color change and 2N HCL was added to stop the reaction. The optical density was measured at 450 nm using a microplate reader (BioTek Epoch) and the results were calculated according to standards.

To identify the candidate cytokines, we used a cytokine array (AbCam) according to the manufacturer’s instructions. Membranes targeting 96 cytokines were incubated with supernatants from control and CAY10566- and T0901317-treated CD4^+^ T cells for 2 h. The membranes were then incubated with a biotinylated antibody cocktail and HRP-conjugated streptavidin for 2 h and 20 min, respectively, followed by washing with buffers I and II. Cytokine expression was measured using a chemiluminescence imaging system (ATTO). The signal intensity was normalized to the positive control spots in the membranes using the ImageJ program (National Institutes of Health, Bethesda, MD, USA; RRID: SCR_003070; http://rsbweb.nih.gov/ij).

### Treatment with cell-free supernatants

To prepare cell-free supernatants from CD4^+^ T cells, we treated the cells with CAY10566 or T0901317 for 1 d. After centrifugation, the supernatant was carefully transferred to a new clear tube to avoid contamination. The supernatant was immediately treated with CD8^+^ T cells and incubated for 1 d. The pre-incubated CD8^+^ T cells were subsequently co-cultured with cancer cells for 1 d after washing with PBS. This procedure is illustrated in Fig. 4B.

### Chemotaxis assay

To evaluate the chemotaxis of CXCR3-CD8^+^ T cells toward CXCL11, CD8^+^ or CXCR3-CD8^+^ T cells were plated in the upper well of a 24-well hanging plate (SPL-Life Science, Pocheon, South Korea), and control or *CXCL11*-siRNA-transfected 4T1 cells were seeded in the bottom well. After 1 day, the number of CD8^+^ T cells was counted after staining with anti-CD8 antibodies. CD8^+^ T cells and recombinant CXCL11 treatment were used as negative and positive controls, respectively.

### Statistical analysis

Experiments were performed independently at least three times. Data are presented as mean ± standard deviation. GraphPad Prism Software (GraphPad Software, San Diego, CA, USA) was used for the statistical analyses. One-way analysis of variance and Tukey’s test (pairwise) were used to compare multiple independent samples. Statistical significance was considered with *P*-values < 0.05.

## Results

### ***CAF-derived lipids increased OA levels in CD4***^+^***TILs ***via*** lipid transporters***

**Fig. 1:**
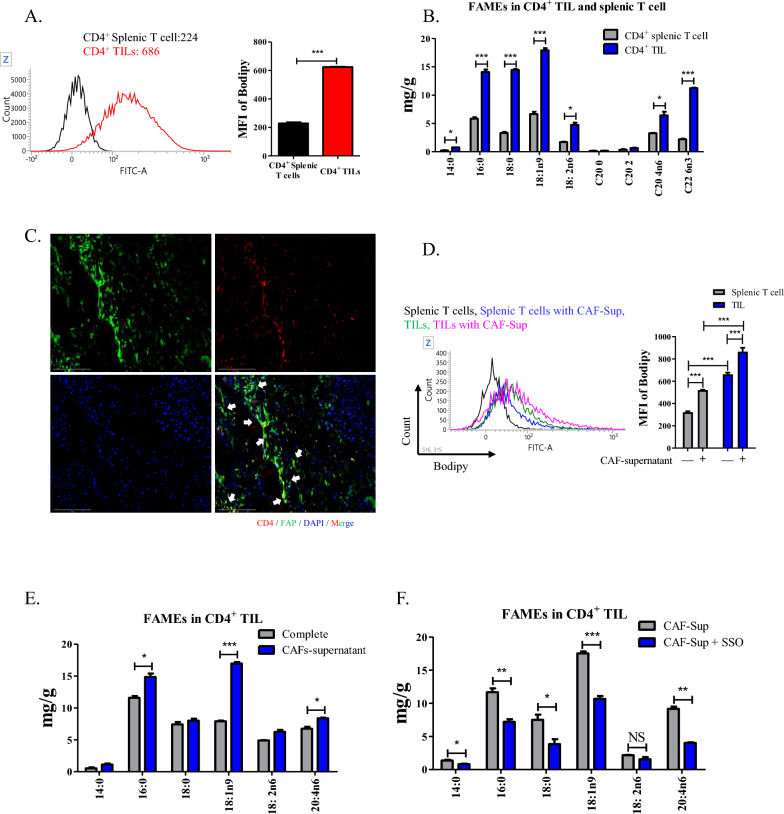
Transition of CAF-derived OA through lipid transporter enhanced lipid metabolism associated markers in CD4^+^ TILs. **A** Representative histogram for BODIPY staining in CD4^+^ splenic T cells and TILs. Black: CD4^+^ splenic T cells, Red: CD4^+^ TILs. The graph indicated the MFI of BODIPY staining. ^***^P < 0.001. Data indicate the mean ± SEM (n = 3). **B** Profile of fatty acid contents in CD4^+^ splenic T cells and TILs. ^*^P < 0.05 and ^***^P < 0.001. Data indicate the mean ± SEM (n = 3). **C** Representative fluorescence image of staining for CD4 and FAP in tumor mass from 4T1 mouse model. Red: CD4, Green: FAP, Blue: DAPI. 40 × magnification. Scale bar: 75 μm. White arrow indicates co-localized fluorescence of CD4 and FAP. **D** Representative histogram for BODIPY staining in CD4^+^ splenic T cells and TILs incubated with CAF-supernatant for 1 day. Black: CD4^+^ splenic T cells, Blue: Treatment of CD4^+^ splenic T cells with CAFs-supernatant, Green: CD4^+^ TILs, Pink: Treatment of CD4^+^ TILs with CAFs-supernatant. The graph indicated the MFI of BODIPY staining. ^***^P < 0.001. Data indicate the mean ± SEM (n = 3). **E** Profile of fatty acid contents in CD4^+^ TILs treated with CAF-supernatant and control. ^*^P < 0.05 and ^***^P < 0.001. Data indicate the mean ± SEM (n = 3). **F** Profile of fatty acid contents in CD4^+^ TILs treated with CAF-supernatant and 50 μM SSO. ^*^P < 0.05, ^**^P < 0.01, and ^***^P < 0.001. Data indicate the mean ± SEM (n = 3)

CD4^+^ tumor-infiltrated lymphocytes (TILs) and splenocytes were isolated from tumor tissue of the 4T1 mouse model and age-matched normal mice (Supplementary fig. S1A). The content of lipid droplets was significantly higher in CD4^+^ TILs than in splenic T cells (Fig. 1A). Further analysis of FAs revealed that the levels of 16:0 (palmitic acid; PA), 18:0 (stearic acid), 18:1n9 (oleic acid; OA), and 22:6n3 (docosahexaenoic acid) were higher in CD4^+^ TILs than in splenic T cells (Fig. 1B). Our previous studies have shown that cancer-associated fibroblasts (CAFs)-derived FA is transported to surrounding tumor cells, resulting in tumor progression [4]. To determine the relationship between CAFs and CD4^+^ TILs, we fluorescently stained CD4 and fibroblast-activation protein (FAP), a marker for CAFs in tumor tissue from 4T1 mouse model. Fluorescence staining showed that CD4^+^ cells were co-localized with FAP (Fig. 1C). However, in spleen, fluorescence for FAP-positive cells did not co-localize with CD4^+^ T cells (Supplementary fig. S1B). To identify whether FAs were transferred from CAFs to CD4^+^ T cells via the cell-free supernatant, both CD4^+^ TILs and splenic T cells were cultured with CAF supernatants for 1 d, followed by evaluation of the lipid and FA content (Supplementary fig. S1C). Incubation of CD4^+^ TILs and splenic T cells with the CAF supernatant significantly enhanced the levels of BODIPY compared those cell incubation with complete media (Fig. 1D). Moreover, FA analysis showed that the levels of OA and PA were upregulated in CD4^+^ TILs after treatment with the CAF supernatant, but the levels of other FAs were unchanged (Fig. 1E). Moreover, incubation with CAFs-supernatant increased the mRNA level of lipid transporter *Cd36* and *Slc27a1* in CD4^+^ TILs compared with control cells (Supplementary fig. S1D). However, treatment with sulfo-*N*-succinimidyl oleate (SSO), a lipid transporter inhibitor, reduced the content of lipid droplet and FAs level in CD4^+^ TILs treated with the CAF supernatant (Fig. 1F and Supplementary fig. S1E). In particular, the level of OA was most significantly reduced compared to other FAs.

### SCD increased the expression levels of Th1 cell markers

**Fig. 2:**
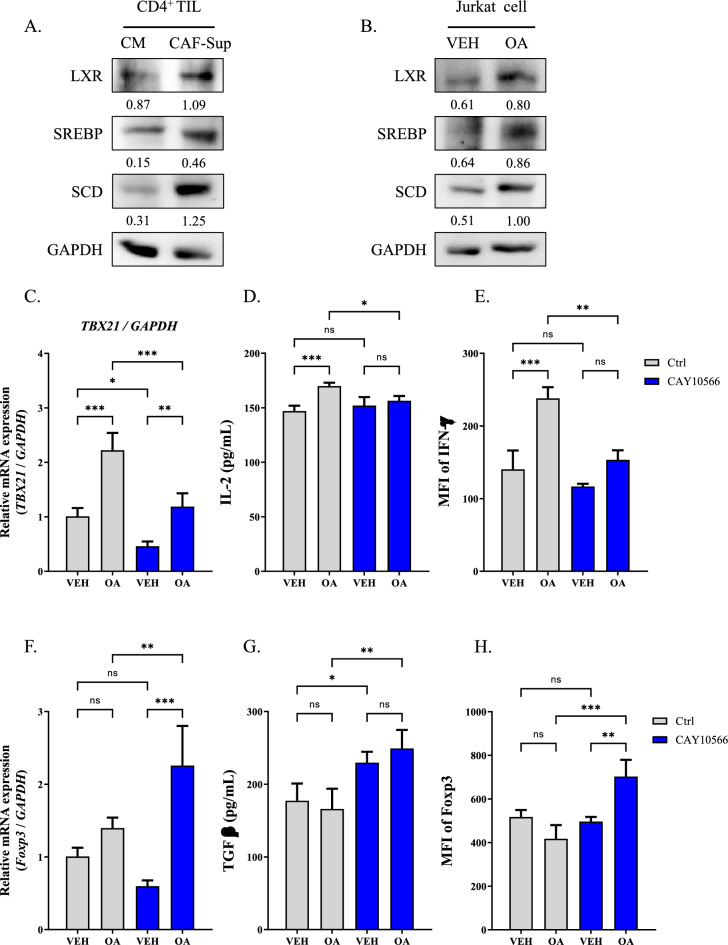
Increase of SCD by OA regulated the expressions of markers for Treg and Th1 cell. **A** Expressions of LXR, SREBP, SCD, and GAPDH in CD4^+^ TILs treated with CAF-supernatant for 1 day. **B** Expressions of LXR, SREBP, SCD, and GAPDH in Jurkat cells treated with 100 μM OA for 1 day. **C**–**E** Evaluation of *TBX21* expression*,* IL-2 concentration, and IFN-γ MFI in Jurkat cells treated with 100 μM OA and 1 μM CAY10566. ^*^P < 0.05. ^**^P < 0.01 and ^***^P < 0.001. Data indicate the mean ± SEM (n = 4). **F**–**H** Evaluation of *FOXP3* expression*,* TGF-β concentration, and FOXP3 MFI in Jurkat cells treated with 100 μM OA and 1 μM CAY10566. ^*^P < 0.05. ^**^P < 0.01 and ^***^P < 0.001. Data indicate the mean ± SEM (n = 4)

We exhibited that incubation with CAF-supernatant enhanced the content of FAs in CD4 + TILs. To determine CAF-supernatant increased the expression of lipid metabolism related molecules, we evaluated the expression of liver-X receptor (LXR), sterol regulatory-element protein (SREBP), and stearoyl-CoA desaturase (SCD) in CD4^+^ TILs after incubation with CAF-supernatant. Incubation with CAF-supernatant upregulated the expressions of LXR, SREBP, and SCD in CD4^+^ TILs compared to those cells incubation with conditioned medium (CM) (Fig. 2A). These results were consistently observed in Jurkat cells after treatment OA (Fig. 2B). CD4 + TILs showed a significantly higher levels of *Tbx21* and *Foxp3*, which are markers of Th1 and Treg cells, respectively. However, no significant difference was observed in the expression levels of *Gata3* or *Rorc*, which are markers associated with Th2 and Th17 cells, respectively (Supplementary fig. S2A). Additionally, treatment of Jurkat cells with CAF-supernatant enhanced the population of positive cells for FoxP3 and T-bet compared to control (Supplementary fig. S2B). Next, to determine the impact of SCD on the expression of Th1 and Treg cell markers, we examined the level of markers of these cells following treatment with CAY10566, an inhibitor of SCD. Treatment with OA augmented the expression levels of markers for both Th1 cell (*TBX21,* IL-2, and IFN-γ) in Jurkat cells compared to the cell cultured in complete medium. However, CAY10566 treatment significantly suppressed the expression levels of *TBX21,* IL-2, and IFN-γ (Fig. 2C–E). Subsequently, we evaluated the level of markers for Treg cell following treatment with OA and CAY10566. Treatment with OA did not alter the levels of *FoxP3* and TGF-β, but combined treatment with CAY10566 significantly increased these levels (Fig. 2F–H).

### Expression of Th1 cell markers were increased dependent on SCD

**Fig. 3:**
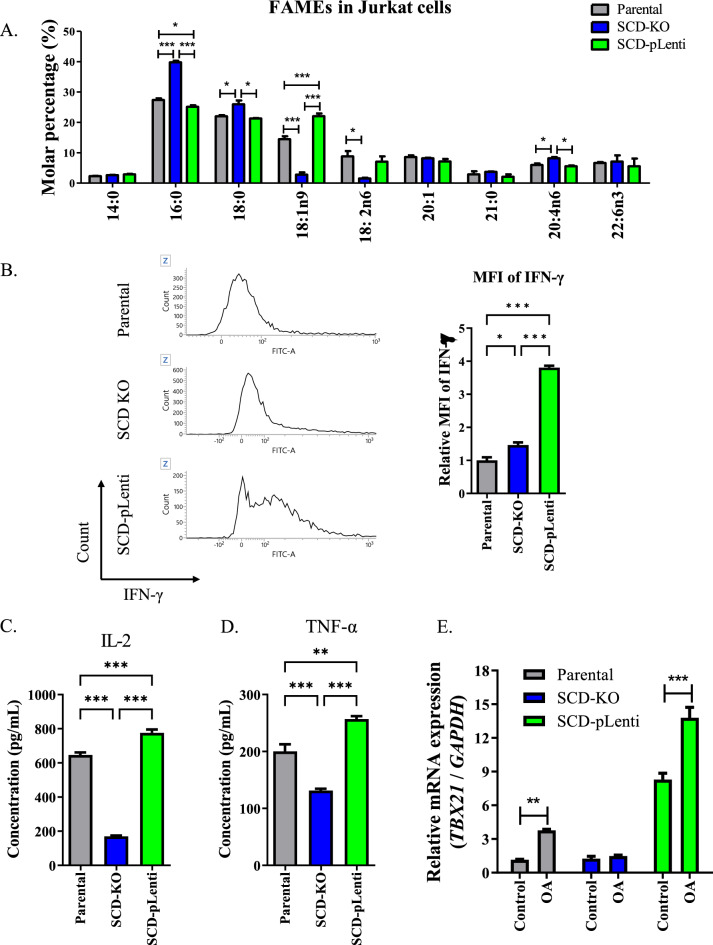
SCD-induced distinctive FA proportion regulated phenotypes of Treg and Th1 cell in Jurkat cells. **A** Profile of FAs molar percentages in SCD subclones of Jurkat cells. ^*^P < 0.05, ^**^P < 0.01 and ^***^P < 0.001. Data indicate the mean ± SEM (n = 3). Gray: parental, Blue: SCD-KO cells, Green: SCD-pLenti cells. **B** Representative histogram of IFN-γ expression in SCD subclones. The graph indicated relative MFI of IFN-γ. ^*^P < 0.05 and ^***^P < 0.001. Data indicate the mean ± SEM (n = 3). **C**, **D** The concentration of IL-2 and TNF-α in SCD subclones. ^**^P < 0.01 and ^***^P < 0.001. Data indicate the mean ± SEM (n = 4). **E** Transcriptional level of *TBX21* in SCD subclones treated with OA. ^**^P < 0.01 and ^***^P < 0.001. Data indicate the mean ± SEM (n = 4)

We produced genetically engineered SCD subclones of Jurkat cells using a pLentiviral vector and CRISPR/Cas9. SCD expression was depleted in SCD-KO cells and overexpressed in SCD-pLenti cells compared to its level in the parental cells and control-Cas9- and pLenti-transfected cells (Supplementary fig. S2C). Analysis of the FA profile revealed a higher proportion of OA in SCD-pLenti cells than in the other subclones, whereas PA level was higher in SCD-KO cells (Fig. 3A). Additionally, the MFI of IFN-γ was higher in SCD-pLenti cells than in other subclones (Fig. 3B). The expression of IL-2 and TNF-α was significantly higher in SCD-pLenti cells compared to the subclones, and their expression was lowest in SCD-KO cells. (Fig. 3C–D). Moreover, treatment with OA which is the highest FAs in SCD-plenti cells enhanced the transcriptional level of TBX21 in parental and plenti cells but did not in SCD-KO cells (Fig. 3E).

### Increase of mitochondrial ROS by PA enhances the expression of Treg cell-associated markers

**Fig. 4:**
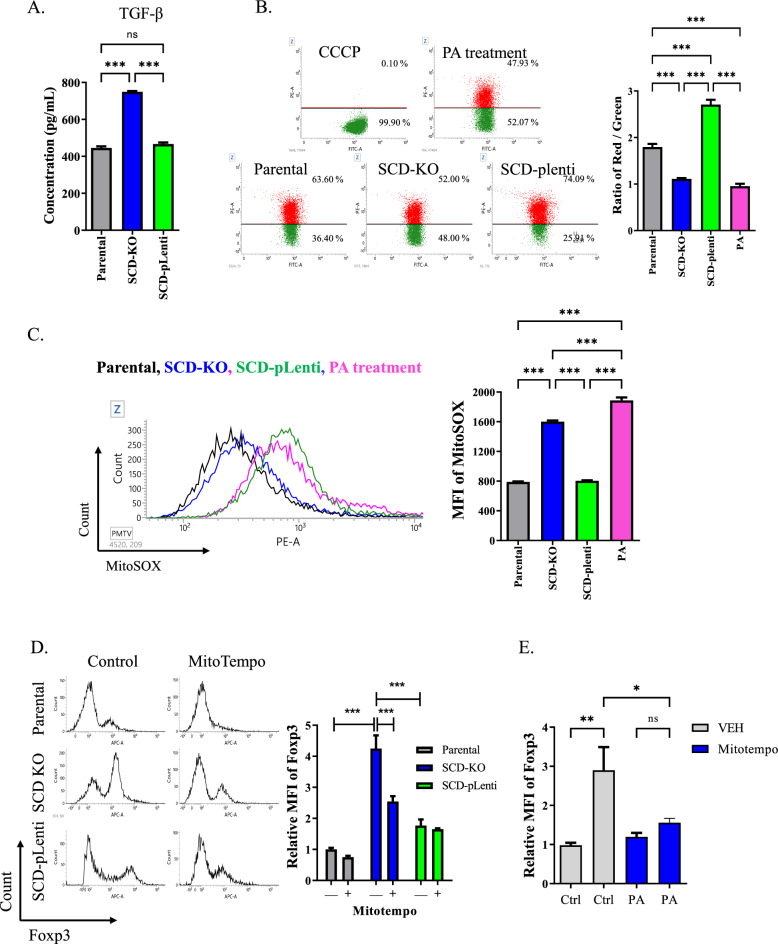
PA in SCD-KO cells enhanced the expression of Treg cell-associated markers via mitochondrial ROS. **A** The concentration of TGF-β in SCD subclones. ^***^P < 0.001. Data indicate the mean ± SEM (n = 4). **B** Representative histogram of MMP in SCD subclones and 100 μM PA treated parental cells. Red and Green portion were divided according to positive control which treated with 50 μM carbonyl cyanide m-chlorophenyl hydrazine (CCCP). The graph indicated the ratio of red to green. ^**^P < 0.01 and ^***^P < 0.001. Data indicate the mean ± SEM (n = 3). **C** Representative histogram for MitoSOX staining in SCD subclones and parental cells treated with100 μM PA. The graph indicated the MFI of MitoSOX staining. ^***^P < 0.001. Data indicate the mean ± SEM (n = 3). Black: Parental, Blue: SCD-KO cells, Green: SCD-pLenti cells, Pink: parental cells treated with PA. **D** Representative histogram for Foxp3 staining in SCD subclones treated with 5 μM mitotempo. The graph indicated the MFI of Foxp3. ^***^P < 0.001. Data indicate the mean ± SEM (n = 4). **E** The graph indicated the relative MFI of Foxp3 in parental Jurkat cells after treatment with 100 μM PA and 5 μM mitotempo. ^*^P < 0.05 and ^**^P < 0.01. Data indicate the mean ± SEM (n = 4)

We observed that expression of TGF-β was enhanced in SCD-KO cells compared to other subclones (Fig. 4A). Moreover, analysis of mitochondrial membrane potential (MMP) exhibited that the ratio of MMP was significantly decreased in SCD-KO cells compared to other subclones (Fig. 4B). To determine the role of PA, which is the highest proportion in SCD-KO cells, we treated the parental cells with PA and evaluated the MMP ratio. Treatment with PA suppressed the ratio of MMP in parental cells. To examine the mitochondrial stress was regulated by SCD, we evaluated the mitochondrial ROS in SCD-subclones by mitoSOX. SCD-KO cells increased the MFI of mitoSOX compared to other subclones. And treatment with PA enhanced the MFI in parental cells (Fig. 4C). We evaluated the expression of Foxp3 in SCD-subclones follwed by chelation of mitochondrial ROS by mitotempo treatment. Foxp3 MFI was increased in SCD-KO cells compared to other subclones. However, its expression was significantly decreased in SCD-KO cells after mitotempo treatment (Fig. 4D). Furthermore, treatment with PA upregulated the MFI of Foxp3 in parental cells, but the MFI was suppressed after chelating the mitochondrial ROS (Fig. 4E).

### ***CXCL11 derived from SCD-upregulated CD4***^+^***T cells enhanced CD8***^+^***T cell activity***

**Fig. 5:**
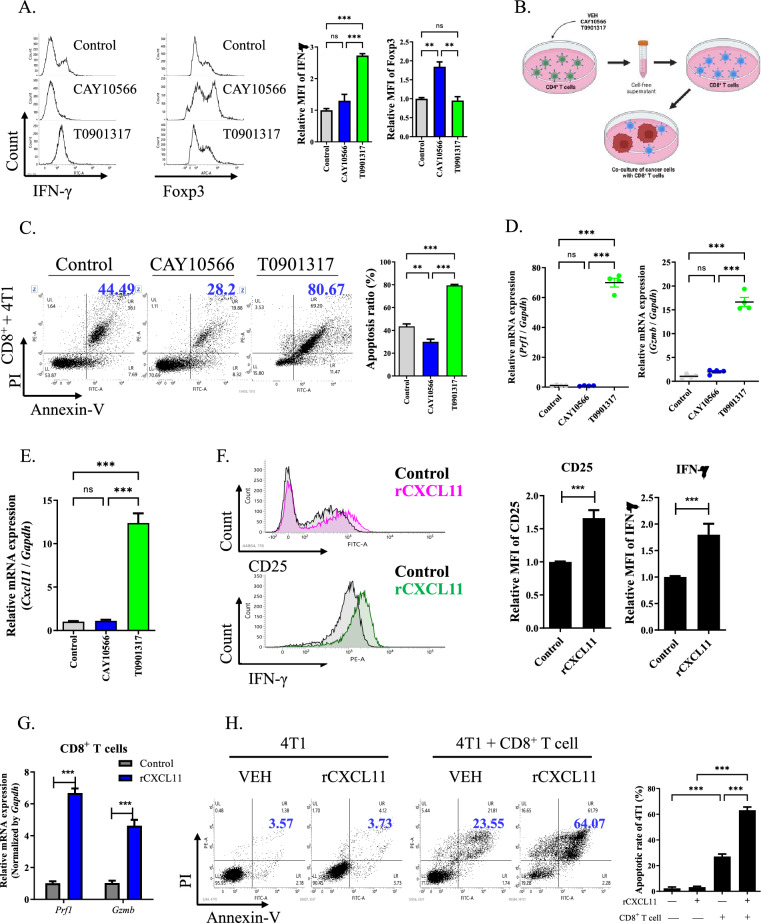
Increase of CXCL11 in CD4^+^ T cells by SCD stimulated activity of CD8^+^ T cells. **A** Representative histograms for IFN-γ and Foxp3 expressions in CD4^+^ splenic T cells treated with 1 μM CAY10566 and 10 μM T0901317 for 1 day. The graph indicated the relative MFI of IFN-γ and Foxp3 expressions. ^**^P < 0.01 and ^***^P < 0.001. Data indicate the mean ± SEM (n = 3). **B** Workflow of co-culture of cancer cells with CD8^+^ T cells that preincubated with cell-free supernatant from CD4^+^ T cells treated with control, 1 μM CAY10566 and 10 μM T0901317 for 1 day. **C** Representative dot plot of staining for annexin-V and PI in 4T1 cells which co-cultured with preincubated CD8^+^ T cells with cell-free supernatant from CD4^+^ T cells treated with control, 1 μM CAY10566 and 10 μM T0901317 for 1 day. The quadrant was divided according to 4T1 cells alone. Apoptotic rate: percentage of upper right + lower right. ^**^P < 0.01 and ^***^P < 0.001. Data indicate the mean ± SEM (n = 3). **D** Transcription levels of *Prf1* and *Gzmb* in CD8^+^ T cells treated with cell-free supernatant from CD4^+^ T cells treated with control, 1 μM CAY10566 and 10 μM T0901317 for 1 day. ^***^P < 0.001. Data indicate the mean ± SEM (n = 4). **E** Transcription level of *Cxcl11* in CD4^+^ T cells treated with 1 μM CAY10566 and 10 μM T0901317 for 1 day. ^***^P < 0.001. Data indicate the mean ± SEM (n = 4). **F** Representative histogram of CD25 and IFN-γ staining in CD8^+^ T cells treated with 10 ng/mL rCXCL11 for 1 day. The graph indicated MFI of CD25 and IFN-γ staining. ^***^P < 0.001. Data indicate the mean ± SEM (n = 3). **G** Transcription levels of *Prf1* and *Gzmb* in CD8^+^ T cells treated with 10 ng/mL rCXCL11 for 1 day. ^***^P < 0.001. Data indicate the mean ± SEM (n = 4). **H** Representative dot plot of staining for annexin-V and PI in 4T1 cells which co-cultured with CD8^+^ T cells treated with 10 ng/mL rCXCL11 for 1 day. The quadrant was divided according to 4T1 cells alone. Apoptotic rate: upper right + lower right. ^***^P < 0.001. Data indicate the mean ± SEM (n = 3)

To further examine the reproducibility of the effect of SCD on mouse CD4^+^ splenic T cells, we analyzed the expression of markers of Th1 and Treg cells, following treatment with an SCD inhibitor and inducer (Supplementary fig. S3A). Upregulation of SCD by T0901317 treatment increased the expression levels of IFN-γ and IL-2 in CD4^+^ splenic T cells, while treatment with an SCD inhibitor enhanced the expression levels of Foxp3 and TGF-β (Fig. 5A, Supplementary fig. S3B). However, the proliferation rate of CD4^+^ splenic T cells was not altered by the inducer or inhibitor treatment (Supplementary fig. S3C). To examine the impact of SCD-regulated CD4^+^ T cells on CD8^+^ T cells, CD8^+^ T cells were pre-incubated with cell-free supernatants from CD4^+^ splenic T cells after treatment with the control, CAY10566, or T0901317 (Supplementary fig. S4A). Subsequently, pre-incubated CD8^+^ T cells were co-cultured with 4T1 cells (Fig. 5B). CD8^+^ T cells preincubated with cell-free supernatant from T0901317-treated CD4^+^ T cells significantly increased the apoptotic rate of 4T1 cells (80.67%) compared to that of the control (44.49%), whereas preincubated CD8^+^ T cells with cell-free supernatant from CAY10566-treated CD4^+^ T cells showed a lower apoptotic rate (28.2%; Fig. 5C). Similar results were obtained for B16F10 and CT26 cells (Supplementary fig. S4B). Treatment of CD8^+^ T cells with T0901317 enhanced the apoptotic rate of 4T1, B16F10, and CT26 cells compared to treatment with the control and CAY10566, but the degree was lower than incubation of co-cultured cells with supernatant from T0901317 treated CD4^+^ T cells (Supplementary fig. S4C). Moreover, pre-incubation of CD8^+^ T cells with cell-free supernatant from T0901317-treated CD4^+^ T cells significantly upregulated the expression levels of *Prf1* and *Gzmb* compared to their levels in control cells (Fig. 5D). An identical pattern was also observed for CD8^+^ T cells treated with T0901317, but to a lesser degree (Supplementary fig. S4D).

Cytokine array analysis revealed that the expression levels of *Cxcl11*, *-13*, and *15*; *Tnfrsf19*; *Igbp3*; *Retn;* and *Igf1* were higher in cell-free supernatants from T0901317-treated CD4^+^ T cells than in those from CAY10566-treated and control cells (Supplementary fig. S4E). A comparable pattern was observed for the transcription levels of the cytokines (Supplementary fig. S4F). Notably, the expression level of *Cxcl11* was enhanced by more than 12-fold in T0901317-treated CD4^+^ T cells compared with its level in control cells (Fig. 5E). Although the levels of other IFN-γ-inducible chemokines, *Cxcl9* and *-10,* increased, their fold change was lower than that of *Cxcl11* (Supplementary fig. S4G). To determine whether CXCL11 regulates CD8^+^ T cell activity, we assessed the activity and apoptosis of 4T1 cells following treatment with rCXCL11. Treatment with rCXCL11 increased the expression levels of markers of CD8^+^ T cell activity, including CD25, IFN-γ, *Prf1*, and *Gzmb* (Fig. 5F, G). Additionally, the treatment of CD8^+^ T cells with rCXCL11 significantly increased the apoptotic rate of 4T1 cells (Fig. 5H).

### ***Conjugation of CXCR3 with CXCL11 enhanced cytotoxic activity of CD8***^+^***T cells***

**Fig. 6:**
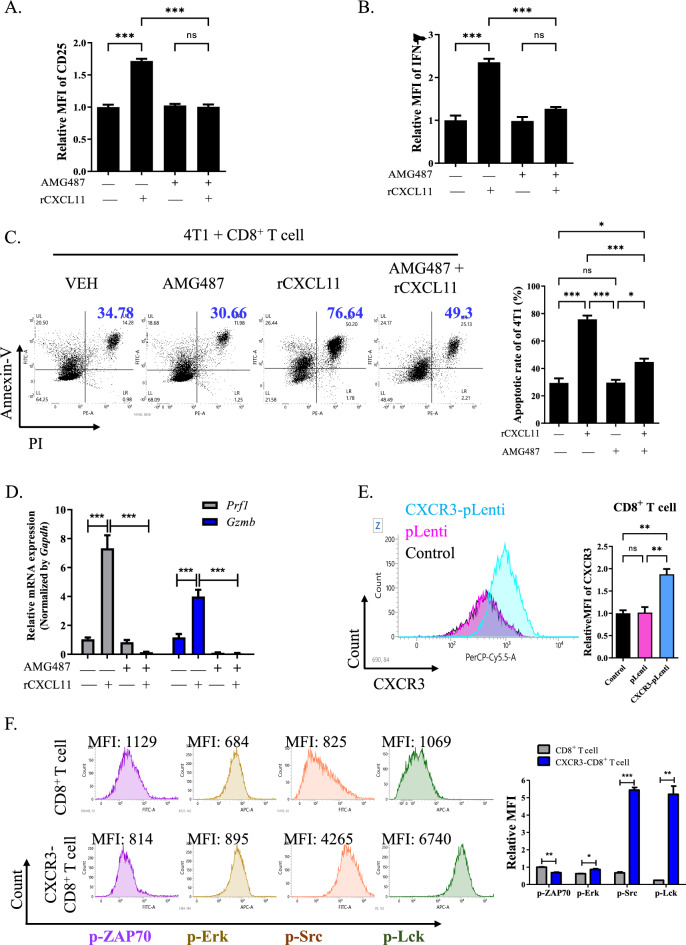
Conjugation of CXCL11 with CXCR3 enhanced cytotoxicity of CD8 + T cells. **A**, **B** Relative MFI of CD25 and IFN-γ in CD8^+^ T cells treated with rCXCL11 and AMG487 for 1 day. ^***^P < 0.001. Data indicate the mean ± SEM (n = 3). **C** Representative dot plot of staining for annexin-V and PI in 4T1 cells which co-cultured with rCXCL11 treated CD8^+^ T cells for 1 day after 18 h pretreatment with 10 nM AMG487. The graph indicated the apoptotic rate of 4T1 cells. Apoptotic rate: upper right + upper left. ^*^P < 0.05 and ^***^P < 0.001. Data indicate the mean ± SEM (n = 3). **D** Transcription levels of *Prf1* and *Gzmb* in CD8^+^ T cells treated with rCXCL11 and AMG487. ^***^P < 0.001. Data indicate the mean ± SEM (n = 4). **E** Representative histogram of CXCR3 staining in CD8^+^ after transduction with pLenti and CXCR3-pLenti. The graph indicated relative MFI of CXCR3 expression. ^**^P < 0.01. Data indicate the mean ± SEM (n = 3). **F** Representative histogram of staining for p-Src, p-Lck, and p-Erk in parental and CXCR3-CD8^+^ T cells. The graphs indicated the MFI of their expressions. ^*^P < 0.05, ^**^P < 0.01 and ^***^P < 0.001. Data indicate the mean ± SEM (n = 3)

To examine the CXCL11 which highly secreted from SCD-upregulated CD4^+^ T cells activated the CD8^+^ T cells dependent on CXCR3, we evaluated the activation markers for CD8^+^ T cells after treatment with rCXCL11 and CXCR3 inhibitor. Treatment with rCXCL11 enhanced the MFI of IFN-γ and CD25. However, combined treatment with AMG487, an antagonist for CXCR3 suppressed the levels of MFI (Fig. 6A, B). In the co-culture of rCXCL11-treated CD8^+^ T cells with 4T1 cells, AMG487 treatment suppressed the CD8^+^-T-cell-induced apoptosis of 4T1 cells (Fig. 6C). However, AMG487 treatment of the 4T1 cells did not increase the rate of apoptosis (Supplementary fig. S5A). A similar pattern was observed for B16F10 and CT26 cells (Supplementary fig. S5B). Furthermore, AMG487 treatment suppressed the expression levels of *Prf1* and *Gzmb* in rCXCL11-treated CD8^+^ T cells (Fig. 6D). Next, we engineered the CXCR3-pLenti CD8^+^ T cells to investigate the role of CXCL11/CXCR3 conjugation in CD8^+^ T cells (Fig. 6E). To investigate the mechanisms underlying CXCL11/CXCR3-axis in CD8^+^ T cells, we evaluated the expression of protein kinases in CXCR3-overexpressing CD8^+^ T cells after treatment with rCXCL11. CXCR3-CD8^+^ T cells showed a significant increase in phosphor (p)-Src and p-Lck levels compared to pLenti-CD8^+^ cells, whereas p-ZAP70 and p-Erk levels did not change (Fig. 6F).

### ***Administration of CXCR3-CD8***^+^***T cells suppressed tumor growth in an immunocompetent mouse model***

**Fig. 7:**
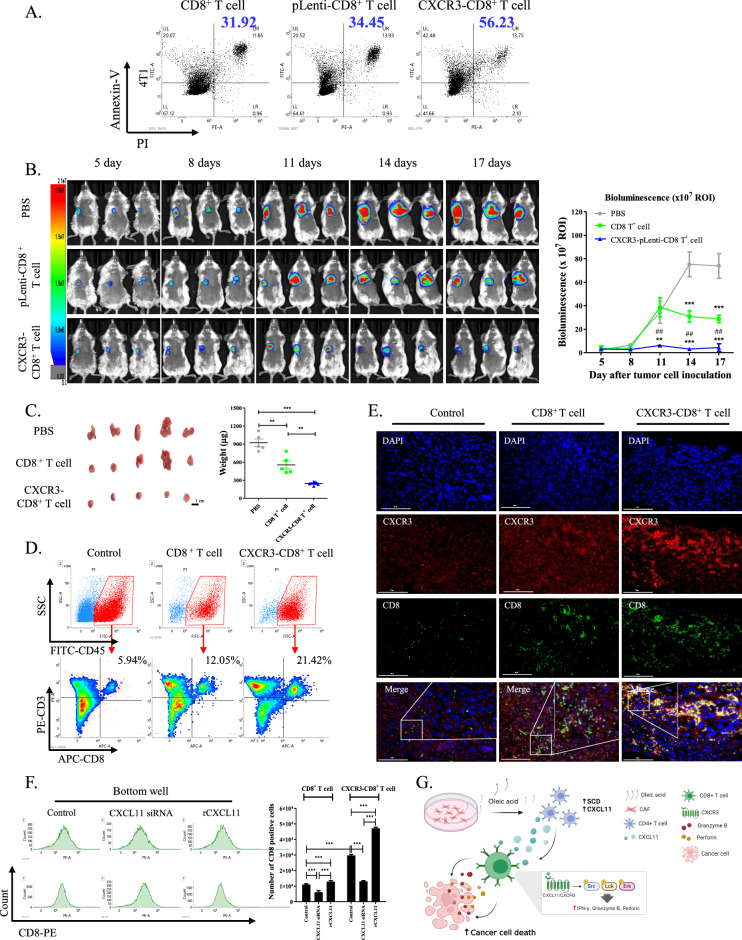
Administration of CXCR3-CD8^+^ T cells abrogates tumor growth in vivo. **A** Representative dot plot of staining for annexin-V and PI in 4T1 cells which co-cultured with pLenti and CXCR3-pLenti CD8^+^ T cells for 1 day. Apoptotic rate: upper right + upper left. **B** The bioluminescence of Luc2-4T1 cells in mouse model was observed for 12 days after administration with PBS, 2 × 10^6^ pLenti-, and CXCR3-CD8^+^ T cells. The bioluminescence intensity was quantified in the mice. PBS vs ^***^P < 0.001. CD8^+^ T cells vs ^*##*^*P* < 0.01. Data indicate the mean ± SEM (n = 5). **C** The size and weight of tumors. Scale bar: 1 cm. ^**^P < 0.01 and ^***^P < 0.001. Data indicate the mean ± SEM (n = 5). **D** Flow cytometric analysis of CD3 and CD8 staining from CD45 positive cells (P1) in TILs. The quadrant was divided according to unstained negative control. **E** Representative fluorescence staining for CD8 and CXCR3 in tumor administrated with PBS, CD8^+^ T cells and CXCR3-CD8^+^ T cells. Green: CD8, Red: CXCR3, Blue: DAPI. 40 × magnification. Scale bar: 75 μm. Enlarged image indicated the co-localization of CD8 and CXCR3. **F** Representative histogram of staining for CD3 in the bottom well. Bottom well: 4T1 cells transfection with control and CXCL11 siRNA. Upper well: CXCR3-CD8^+^ T cells. As positive control, rCXCL11 was treated in parental CD8^+^ T cells. The graph indicated the number of CD8 positive cells in bottom well. ^***^P < 0.001. Data indicate the mean ± SEM (n = 3). **G** Graphical abstract for interaction of CAFs, CD4^+^ T cells, and CD8^+^ T cells

Treatment with rCXCL11 significantly enhanced the apoptotic rate of 4T1 cells (56.23%) compared to that of parental (31.92%) and pLenti-CD8^+^ T cells (34.45%; Fig. 7A). Similar results were obtained for B16F10 and CT26 cells (Supplementary fig. S6B). Next, we assessed the antitumor effects of CXCR3-CD8^+^ T cells in vivo. The administration of CXCR3-CD8^+^ T cells significantly suppressed tumor growth in the 4T1 mouse model (Fig. 7B). Administration of pLenti-CD8^+^ T cells also reduced tumor growth compared to PBS, but the antitumor efficacy was lower than that achieved by the administration of CXCR3-CD8^+^ T cells. The volume and weight of tumors were reduced in mice treated with CXCR3-CD8^+^ T cells compared to those treated with PBS and pLenti-CD8^+^ T cells (Fig. 7C). Additionally, the population of tumor-infiltrating CD8^+^ T cells was higher in mice treated with CXCR3-CD8^+^ T cells (21.42%) than in those treated with PBS (5.94%) or pLenti-CD8^+^ T cells (12.05%; Fig. 7D). The expression level of IFN-γ was higher in tumor-infiltrating CD8^+^ cells isolated from tumors of mice administered CXCR3-CD8^+^ T cells compared its expression level in PBS and pLenti-CD8^+^ T cells injected mice (Supplementary fig. S6C). Furthermore, CD8 fluorescence colocalized with CXCR3 in the tumors of mice treated with CXCR3-CD8^+^ T cells (Fig. 7E). Based on these findings, we examined CXCR3/CXCL11-axis-mediated chemotactic activity in cancer and CD8^+^ T cells. The migration of CXCR3-CD8^+^ T cells to 4T1 cells was substantially higher than that the migration of pLenti-CD8^+^ T cells to 4T1 cells. However, migration was suppressed when CXCR3-CD8^+^ T cells were co-cultured with CXCL11-silenced 4T1 cells (Fig. 7F). In vitro and in vivo results illustrated that CAF-derived oleic acid enhanced SCD expression and secretion of CXCL11 in CD4^+^TILs. In addition, binding of CXCL11 with CXCR3 on CD8^+^ T cells enhanced the killing activity to cancer cells (Fig. 7G). In addition, our co-culture system revealed that the treatment of pLenti- and CXCR3-CD8^+^ T cells with an anti-PD1 antibody increased the apoptotic rate of 4T1 cells (Supplementary fig. S6D). To examine the synergistic effect in the 4T1 mouse model, we administered an anti-PD1 antibody with PBS, pLenti-, or CXCR3-CD8^+^ T cells (Supplementary fig. S6E). Combined therapy of the anti-PD1 antibody with PBS and CD8^+^ T cells showed a synergistic effect compared to single administration; however, this effect was not observed in combination with CXCR3-CD8^+^ T cells (Supplementary fig. S6F–H).

## Discussion

Immune responsiveness is highly activated in TILs compared to normal lymphocytes. Adoptive TIL transfer therapy provides clinical benefits, with a durable complete response in patients [25, 26]. However, the tumor microenvironment constrains the therapeutic potential of this therapy by suppressing immunosurveillance and promoting the aggressive transformation of cancer cells. Additionally, the tumor microenvironment induces metabolic alterations in cancerous and noncancerous cells. Recent studies have focused on the role of lipid metabolism, which provides energy for uncontrolled proliferation, oncogenic signaling, and the evasion of apoptosis. Our previous study revealed that CAF-derived FAs in cell-free supernatants enhance cancer cell stemness [4]. However, the comprehensive role of the FAs secreted by CAFs in CD4^+^ T cells remains poorly understood. CAF secrets high amounts of lipid that transfers to neighboring cancer cells. In this study, we demonstrated that the transfer of OA from CAF to CD4^+^ TILs via supernatant increased lipid metabolism activation and favored polarization toward Th1 cell over Treg cell. Among lipid metabolism-associated molecules, SCD expression was significantly enhanced in CD4^+^ TILs following treatment with CAF-supernatant. We discovered that increased SCD in CD4^+^ T cells promotes Th1 cell polarization and activation of the cytotoxic effect in CD8^+^ T cells within the tumor immune microenvironment.

Our previous study revealed that lipid metabolic interaction of CAF and cancer cells promotes tumor progression by SCD-mediated stemness in cancer cells [4]. However, the impact of lipid metabolic interaction of CAF and CD4^+^ T cells remain unclear. Our study demonstrated that activation of lipid metabolism in CD4^+^ T cells by CAF-supernatant enhanced SCD expression and favored polarization toward Th1 cell over Treg cell. We discovered that SCD in CD4^+^ T cells promote Th1 cell polarization and activation of the cytotoxic effect in CD8^+^ T cells within the tumor immune microenvironment.

In patients with solid tumor, SCD enhances chemoresistance, cancer stemness, EMT, thereby promoting poor prognosis. Conversely, SCD in CD4^+^ T cells favors polarization toward Th1 cell over Treg cell [27–29]. Notably, in patients with breast cancer, a group with high *CD4* and high *SCD* levels showed a longer DFS compared to a group with high *CD4* and low *SCD* levels (Supplementary fig. S7A, B). Additionally, in patients with lymphoma, a group with high *SCD* levels exhibited better prognosis compared to a group with low *SCD* levels (Supplementary fig. S7C, D). Consistent with this, our study illustrated that SCD promotes Th1 cell polarization in CD4^+^ T cells over Treg cell. SCD-overexpressing Jurkat cells exhibited high levels of OA and Th1 cell markers. Treatment of parental Jurkat cells with OA enhanced the expression levels of Th1 cell markers. However, treatment with PA, an abundant FA in SCD-KO cells, augmented mtROS levels and increased expression of Treg cell markers. These findings indicate that a biased FA proportion may regulate the expression of Th1 and Treg cell markers.

Furthermore, an increase in CXCL11 by SCD-overexpressing CD4^+^ T cells enhanced the recruitment and activation of cytotoxic effect in CD8^+^ T cells via binding with CXCR3. Activation of the CXCL11/CXCR3-axis in CD8^+^ T cells significantly increased apoptosis of cancer cells and reduced tumor growth both in in vitro and in vivo. These findings suggested that an increase of SCD in CD4^+^ T cells, induced by CAF-derived OA, enhanced Th1 cell polarization and CXCL11 secretion, promoting CD8^+^ T cell-mediated tumor suppression via activation of CXCL11/CXCR3-axis.

In the tumor microenvironment, reprogrammed lipid metabolism plays a crucial role in improving the efficacy of immunotherapy by upregulating IFN-γ and granzyme B in mouse melanoma models [30]. FAs contribute to the expansion and differentiation of CD4^+^ T cells [31]. Indeed, SFA treatment suppressed the proliferation and activation of T cells, which was ameliorated by UFA treatment [32]. Additionally, ligation of exogenous OA with LXR augments SCD expression and activates FA metabolism, providing fuel for cell proliferation [33, 34]. In our study, treatment with CAF supernatant increased the amount of lipid droplets in both CD4^+^ TILs and splenocytes, particularly enhancing the proportion of oleic acid among the fatty acids. However, this increase was reduced after the inhibition of the lipid transporter. Additionally, treatment with CAF supernatant enhanced the expression of lipid metabolism-associated molecules and the markers for Th1 and Treg cells. In the tumor microenvironment, CAFs could induce supernatant-mediated metabolic reprogramming of immune cells, thereby controlling their polarization. Moreover, our study showed increased SCD levels by CAF-derived OA was found to enhance the expression levels of Th1 cell markers. SCD-overexpression in CD4^+^ T cells increased the amount of OA to a greater extent than the other FAs. Furthermore, a growing body of evidence indicates that a diet containing a high amount of OA increases the expression levels of Th1 cell markers, including IFN-γ and IL-2, in CD4^+^ T cells [35] and the levels of *CXCL9*, *10*, and *11* [36]. Treatment with CXCL11 induces the differentiation of CD4^+^ T cells into Th1 cells, rather than into Th2 and Th17 cells [37]. In line with these findings, we found that SCD-overexpressing cells showed increased CXCL11 expression level compared to the other subclones. Thus, our data indicated that increased SCD expression levels in CD4^+^ TILs enhanced the expression levels of Th1 cell markers via an OA.

Compared to other subclones, SCD-KO CD4^+^ T cells showed higher levels of Treg cell markers and a higher proportion of PA. In immune cells, conjugation of PA with toll-like receptor 4 activates inflammatory signaling and mitochondrial stress, and suppresses the secretion of TNF-α, IFN-γ, and granzyme B [38, 39]. Consistent with these results, PA treatment enhanced the expression levels of Treg cell markers and mitochondrial superoxide levels. However, superoxide scavenging by MitoTEMPO suppressed Foxp3 expression levels. Our data provide compelling evidence that the increase in PA in SCD-KO mice, achieved by blocking its conversion to monounsaturated fatty acid (MUFA), results in notable upregulation of Treg cell markers through mitochondrial stress. These findings suggest that a biased FA proportion exerts a distinctive effect on the differentiation of CD4^+^ T cell subsets.

Recent studies have highlighted that SCD plays a multifaceted role by regulating the lipid-mediated inflammatory response, promoting the biosynthesis of MUFA and influencing the production of cytokines by T cells. SCD-induced mitochondrial fatty acid oxidation is necessary to supply the energy in cancer cells. SCD plays a pro-tumor role in cancer cells by enhancing stemness, EMT, and chemoresistance. The conversion of saturated fatty acids to unsaturated fatty acids by SCD provides energy that boosts proliferation and reduces oxidative stress, thereby promoting tumor progression. Consequently, inhibition of SCD suppresses tumor growth by increasing apoptosis and ferroptosis [40]. In CD4^+^ T cells, SCD plays a crucial role in differentiation. SCD inhibition enhances the differentiation of CD4^+^ T cells toward Treg cells by increasing the pro-inflammatory response. SCD KO in CD4^+^ T cell enhances a severe inflammatory response leading to enlargement of the spleen and large intestine, as well as mucosal proliferation and thickening [27, 28]. Additionally, increased adipose triglyceride lipase (ATGL) by SCD-KO promotes differentiation of CD4^+^ T cells into Treg cells through a pro-inflammatory response mediated by docosahexaenoic acid [29]. These findings suggest that SCD inhibits Treg cell differentiation by suppressing the pro-inflammatory response and increasing the conversion of fatty acids to unsaturated fatty acid in CD4^+^ T cells.

In contrast, the increase in SCD levels caused by T0901317 treatment enhanced the anti-inflammatory response by downregulating IL-6 and IL-8 secretion [41]. Our study illustrated that SCD upregulation in CD4^+^ T cells significantly increased the secretion of CXCL9, 10, and 11 compared to their secretion by SCD-downregulated and parental cells. Among these, CXCL11 has the highest binding affinity for CXCR3 [20]. Binding of CXCL11 to CXCR3 stimulates the expansion, activation, and migration of CD8^+^ T cells [42]. Consistent with the findings of a previous study, we observed that the binding of CXCL11 to CXCR3 on CD8^+^ T cells enhanced the apoptotic rate of cancer cells. Notably, a recent clinical study reported that adoptive CD8^+^ T-cell therapy has a promising response, with longer progression-free survival, in patients with cancer [26]. To validate these findings, we conducted CD8^+^ T cell adoptive therapy using a mouse tumor model. CXCR3-CD8^+^ T cell treatment significantly suppressed tumor growth by harnessing chemotaxis via the CXCR3/CXCL11 axis in 4T1 mouse models. However, co-delivery of the anti-PD1 antibody with CXCR3-CD8^+^ T cells did not result in significant tumor suppression, whereas combined therapy with CD8^+^ T cells resulted in a substantial reduction in tumor growth. We speculated that a single injection of CXCR3-CD8^+^ T cells was sufficient to reduce tumor growth in our 4T1-mouse model, whereas the addition of anti-PD1 was necessary to induce the efficacy of CD8^+^ T cells.

CXCL11 is highly secreted by immune cells and possesses the highest binding affinity for CXCR3 compared to CXCL9 and CXCL10. In cancer cells, the binding of CXCL11 to CXCR3 enhances EMT and cancer stemness, promoting tumor progression. Conversely, inhibition of CXCR3 suppresses the migration of cancer cells and expressions of EMT-related molecules [4, 43, 44]. However, in naïve CD4^+^ T cells, binding of CXCL11 to CXCR3 induces Th1 differentiation, enhancing CD8^+^ T cells activation through increased secretion of IFN-γ and IL-2. Additionally, the CXCL11/CXCR3 axis augments the recruitment of immune cells, including CD8^+^ T cells, natural killer cells, and macrophages, in in vivo models [20]. Moreover, CXCR3 KO in CD8^+^ T cells reduce their activation and cytotoxic capacity, thereby accelerating tumor growth compared to the wild type. Adoptive transfer of CXCR3 KO CD8^+^ T cells did not inhibit tumor growth or infiltration, nor did it respond to anti-PD-1 antibody treatment [45]. Therefore, we did not inject CXCL11 recombinant protein or CXCR3 inhibitors in our mouse model, primarily due to contradictory role of CXCL11/CXCR3-axis depending on the cell types. The iSCD^−/−^ mouse model, specifically targeting intestinal epithelium, exhibited greater tumor tissue growth compared to the iSCD^+/+^ model. However, dietary supplementation with OA mitigated inflammation and reduced tumor development [46]. SCD plays a different role dependent on the cell lineage and tumor contexts. In the breast cancer cohort (n = 1100) from TCGA Firehose Legacy datasets, data of 533 patients (48.45%) who had *CD4* high were included (Suppl. Table S2). Among them, 245 patients (45.97%) and 244 patients (45.78%) had a high and low *SCD* gene expression, respectively. In the *CD4* high breast cancer cohort (n = 533), SCD expression data were not available for 44 patients (8.26%). In a subgroup of high *CD4* (n = 533), patients with high *SCD* had significantly higher disease-free survival [DFS; median 214.72 (95% confidence interval, 107.98 – NA)] months compared to patients with low *SCD* (Supplementary fig. S7A). However, 567 patients (51.55%) who had low *CD4* in the breast cancer cohort (n = 1100) (Suppl. Table S2) showed a non-significant difference in DFS between high *SCD* (n = 253, 44.62%) and low *SCD* (n = 255, 44.97%) (Supplementary fig. S7B). In the *CD4* low breast cancer cohort (n = 567), SCD expression data were not available for 59 patients (10.41%). Additionally, in chronic myeloid leukemia (CML) mouse model, SCD attenuates the development of CML and enhances the response to anticancer drug [47]. In the B-cell lymphoma cohort (n = 48) from TCGA Firehose Legacy datasets (Suppl. Table S3), although significance was not observed, group with high *SCD* expression showed longer overall survival (OS) and DFS rate than group with low *SCD* expression (Supplementary fig. S7C-D). The manipulation of SCD expression by injection with its inhibitor was limited in mouse model because systemic circulation. Therefore, we did not modulate the SCD expression and instead focused on the modulation of CXCL11/CXCR3-axis, a consequence of the underlying mechanism of SCD-overexpressed CD4^+^ T cells.

## Conclusions

In conclusion, our study provides mechanistic insights into the effect of SCD-dependent FAs on the differentiation of CD4^+^ T cells and proposes a prospective strategy for enhancing antitumor immunity through the CXCL11/CXCR3 axis in CD8^+^ T cells.

## Supplementary Information

Supplementary figure S1. Characterization of lipid metabolism in TILs incubated with CAFs-supernatant. (A) Representative dot plot image of CD3 and CD4 staining in isolated CD4+ splenocytes and TILs in tumor tissue isolated from 4T1 mouse model. (B) Representative fluorescence image of staining for CD4 and FAP in spleen from normal mouse. Red: CD4, Green: FAP, Blue: DAPI. Scale bar: 100 μm. (C) Representative histogram of FAP staining in isolated CAFs from tumor mass in 4T1 cells. The P4 population was established using negatively isolated cells. ***P<0.001. Data indicate the mean ± SEM (n = 3). (D) Transcription levels of Cd36 and Slc27a1 in CD4+ splenic T cells and TILs incubated with CAF-supernatant for 1 day. *P < 0.05 and ***P < 0.001. Data indicate the mean ± SEM (n = 4). (E) Representative histogram of BODIPY staining in CD4+ TILs treated with CAF-supernatant and SSO. The graph indicated the MFI of BODIPY staining. ***P<0.001. Data indicate the mean ± SEM (n = 3). Supplementary figure S2. SCD regulates the expressions of marker for Treg and Th1 cells and mitochondrial function. (A) Transcription levels of Tbx21, Gata1, Rorγ, and Foxp3 in CD4+ splenic T cells and TILs. **P<0.01 and **P<0.01. Data indicate the mean ± SEM (n = 4). (B) Analysis of FoxP3 and T-bet staining in Jurkat T cells after treatment with CAF-supernatant for 1 day. (C) Expressions of SCD and GAPDH in parental, Control-Cas9-transfected cells, SCD-KO cells, empty-pLenti-infected cells and SCD-pLenti cells. Supplementary figure S3. The effect of SCD in CD4+ splenic T cells. (A) Expression of SCD in CD4+ splenic T cells treated with control, 1 μM CAY10566 and 10 μM T0901317 for 1 day. (B) The concentration of TGF-β and IL-2 in CD4+ splenic T cells treated with control, 1 μM CAY10566 and 10 μM T0901317. **P < 0.01 and ***P < 0.001. Data indicate the mean ± SEM (n = 4). (C) CFSE staining in CD4+ T cells treated with control, 1 μM CAY10566 and 10 μM T0901317 for 1 day. The fluorescent of CFSE staining was measured for 3 days. The graph indicated the MFI of CFSE staining. Data indicate the mean ± SEM (n = 4). Supplementary figure S4. Increase of CXCL11 secretion by SCD augmented the activity of CD8+ T cells dependent on binding with CXCR3. (A) Representative dot plot image of CD3 and CD8 staining in isolated CD8+ T cells from splenocytes. (B) Representative dot plot of staining for annexin-V and PI in B16F10 and CT26 cells which co-cultured with preincubated CD8+ T cells with cell-free supernatant from control, 1 μM CAY10566, and 10 μM T0901317-treated CD4+ T cells. The quadrant was divided according to untreated B16F10 and CT26 cells. Apoptotic rate: upper right + lower right. (C) Representative dot plot of staining for annexin-V and PI in 4T1, B16F10 and CT26 cells which co-cultured with CD8+ T cells treated with 1 μM CAY10566 and 10 μM T0901317 for 1 day. Apoptotic rate: upper right + lower right. (D) Transcription levels of Prf1 and Gzmb in CD8+ T cells treated with 1 μM CAY10566 and 10 μM T0901317. *P < 0.05, **P < 0.01, and ***P < 0.001. Data indicate the mean ± SEM (n = 4). (E) The membrane of cytokine array using supernatant from CD4+ T cells treated with control, 1 μM CAY10566 and 10 μM T0901317. (F) Transcription levels of Cxcl13, Cxcl15, Fgf2, Tnfrsf19, Igfbp3, Retn and Igf1 in CD4+ splenic T cells treated with control, 1 μM CAY10566 and 10 μM T0901317. *P < 0.05, **P < 0.01 and ***P < 0.001. Data indicate the mean ± SEM (n = 4). (G) Transcription levels of Cxcl9 and 10 in CD4+ T cells treated with control, 1 μM CAY10566 and 10 μM T0901317. Data indicate the mean ± SEM (n = 4). (K) Representative dot plot of staining for annexin-V and PI in B16F10 and CT26 cells co-cultured with preincubated CD8+ T cells with rCXCL11 and AMG487 for 1 day. The quadrant was divided according to untreated B16F10 and CT26 cells Apoptotic rate: upper right + lower right. Supplementary figure S5. CXCL3/CXCL11-axis augmented activity of CD8+ T cells. (A) Representative dot plot of staining for annexin-V and PI in 4T1 cells treated with 20 μM AMG487 for 18 hours. The quadrant was divided according to untreated 4T1 cells. Apoptotic rate: upper left + upper right. (B) Representative dot plot of staining for annexin-V and PI in B16F10 and CT26 cells co-cultured with alone, parental and CXCR3-CD8+ T cells. The quadrant was divided according to untreated B16F10 and CT26 cells. Apoptotic rate: upper right + lower right. Supplementary figure S6. Single administration of CXCR3-CD8+ T cells suppressed tumor growth in mouse model. (A) Schedule of 4T1-mice model injected with PBS, CD8+ T cells, and CXCR3-CD8+ T cells for 17 days. (B) Representative dot plot of staining for annexin-V and PI in CT26 and B16F1 cells which co-cultured with parental, pLenti- and CXCR3-CD8+ T cells. Apoptotic rate: upper right + lower right. (C) Representative histogram of staining for IFN-γ in CD8+ TILs which isolated from PBS, CD8+ T cells and CXCR3-CD8+ T cells injected mouse. The graph indicated the MFI of IFN-γ. **P < 0.01. Data indicate the mean ± SEM (n = 5). (D) Representative dot plot of staining for annexin-V and PI in 4T1 cells which co-cultured with 1 μg/mL anti-PD1 antibody treated CD8+ T cells and CXCR3-CD8+ T. Apoptotic rate: upper right + lower right. (E) Schedule of 4T1-mice model injected with PBS, CD8+ T cells, and CXCR3-CD8+ T cells and anti-PD1 antibody for 17 days. (F) The bioluminescence of Luc2-4T1 cells in mouse model was monitored for 17 days following injections with PBS, 2×106 CD8+ T cells and CXCR3-CD8+ T cells and 200 μg of anti-PD1 antibody for 3 times. (G) The graph indicated bioluminescence intensity in the mice model. PBS vs ***P < 0.001. #P < 0.05 and #P < 0.001. Data indicate the mean ± SEM (n = 5). (I) Measurement of tumor volume for 17 days in 4T1 mouse model which injected with 200 μg anti-PD1 antibody and PBS, CD8+ T cells and CXCR3-CD8+ T cells. PBS vs ***P < 0.001. #P < 0.05 and ##P < 0.01. Data indicate the mean ± SEM (n = 5). (H) The weight of tumors separated from mice injected with anti-PD1 antibody and PBS, CD8+ T cells, and CXCR3-CD8+ T cells. *P<0.05, **P<0.01, and ***P < 0.001. Data indicate the mean ± SEM (n=5). Supplementary figure S7. Analysis of clinical prognosis dependent on SCD in TCGA data sets. Analysis of Kaplan-Meier curve for DFS in patients with breast cancer (n=1100) who had high CD4 expression (n=533, 48.45%) and low CD4 expression (n=567, 51.55%). (A) Among high CD4 expression patients, Kaplan-Meier curve for DFS in patients with high SCD expression (n=245, 45.97%) and low SCD expression (n=244, 45.78%). P value: 0.0260. (B) Among low CD4 patients, Kaplan-Meier curve for DFS in patients with high SCD expression (n=253, 44.62%) and low SCD expression (n=255, 44.97%). P value: 0.3501. Analysis of Kaplan-Meier curve for overall survival (OS) and DFS in patients with B-cell lymphoma (n=48). (C) Kaplan-Meier curve for OS in patients with high SCD expression (n=25, 52.08%) and low SCD expression (n=23, 47.92%). P value: 0.500. (D) Kaplan-Meier curve for DFS in patients with SCD high expression (n=24, 50.00%) and SCD low expression (n=20, 41.67%). P value: 0.587. In the DFS data (n = 48), SCD expression data were not available for 4 patients (8.33%).

Supplementary Material 2.

Supplementary Material 3.

Supplementary Material 4.

## Data Availability

The data are available when request to the corresponding author.

## Abbreviations

CAF: Cancer-associated fibroblasts
CXCL: C–X–C motif chemokine ligand
CXCR3: C–X–C chemokine receptor 3
DCF-DA: 2,7-Dichlorodihydrogluorescein diacetate assay
FA: Fatty acid
FAP: Fibroblast activation protein
HRP: Horseradish peroxidase
IFN-γ: Interferon-γ
IL-2: Interleukin-2
KO: Knockout
LXR: Liver X receptor
MFI: Mean fluorescence intensity
MMP: Mitochondrial membrane potential
mtROS: Mitochondrial reactive oxygen species
OA: Oleic acid
PA: Palmitic acid
PBS: Phosphate-buffered saline
PBS-T: Phosphate-buffered saline with 0.1% Tween 20
PCR: Polymerase chain reaction
ROS: Reactive oxygen species
RPMI: Roswell Park Memorial Institute
SCD: Stearoyl-CoA desaturase
SFA: Saturated fatty acid
SREBP: Sterol regulatory-element protein
SSO: Sulfosuccinimidyl oleate
TGF-β: Transforming growth factor-β
TIL: Tumor-infiltrating lymphocyte
TNF-α: Tumor-necrosis factor-α
Treg: Regulatory T
UFA: Unsaturated fatty acid
