# Supplementary figures and images for: Stearoyl-CoA desaturase in CD4+ T cells suppresses tumor growth through activation of the CXCR3/CXCL11 axis in CD8+ T cells
Source: Cell Biosci. 2024 Nov 14;14:137. doi: 10.1186/s13578-024-01308-3 (PMC11566202; doi:10.1186/s13578-024-01308-3)

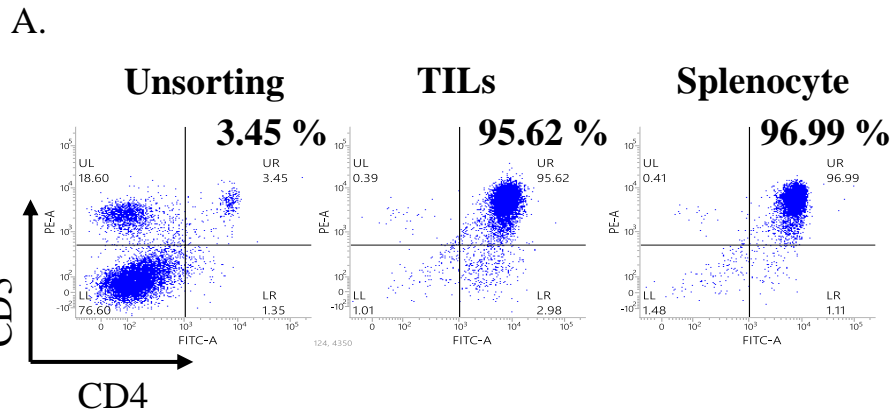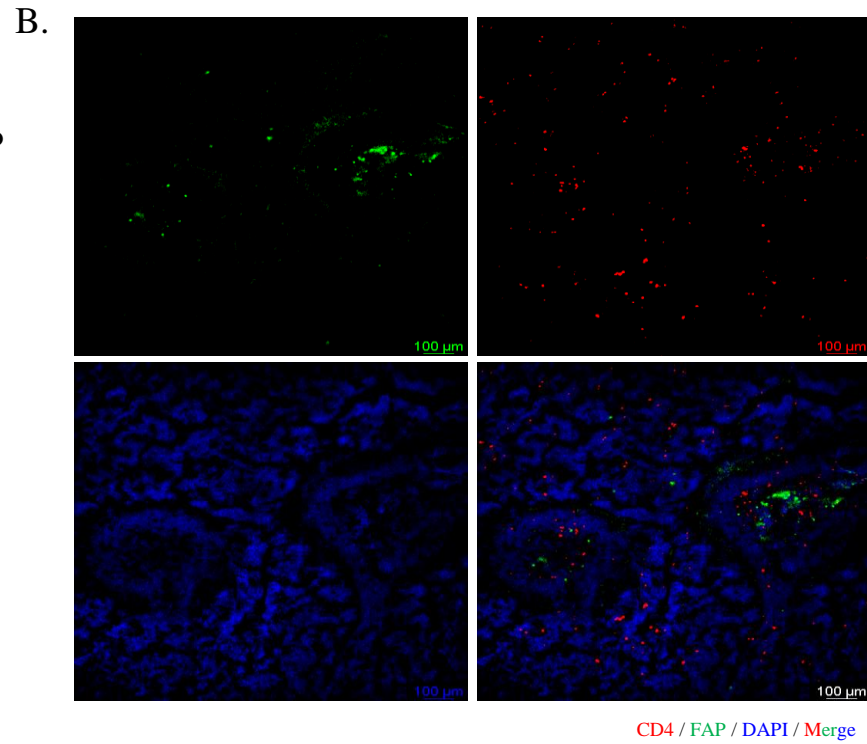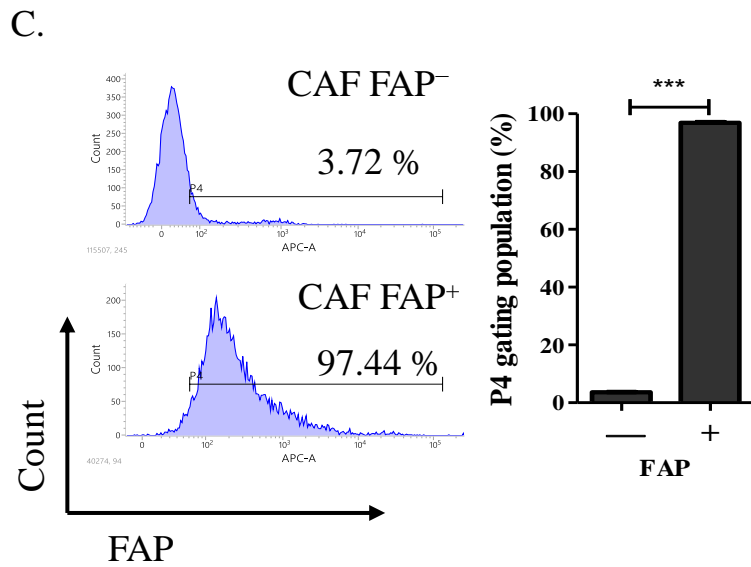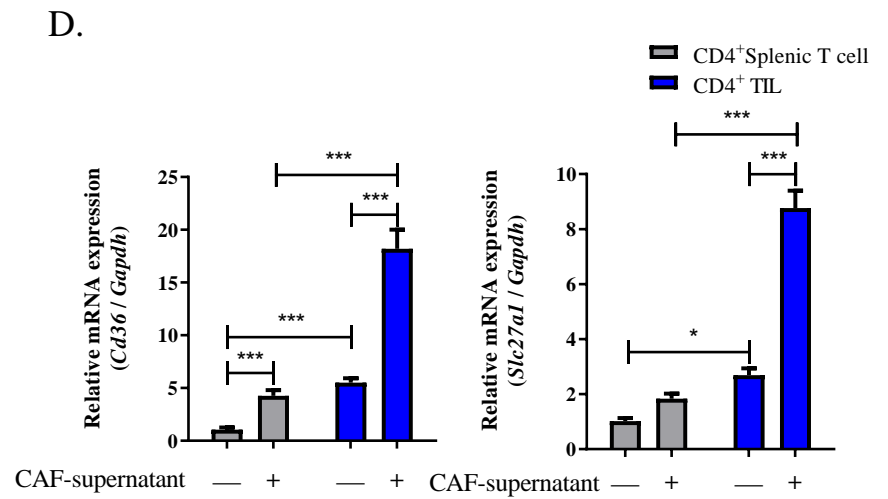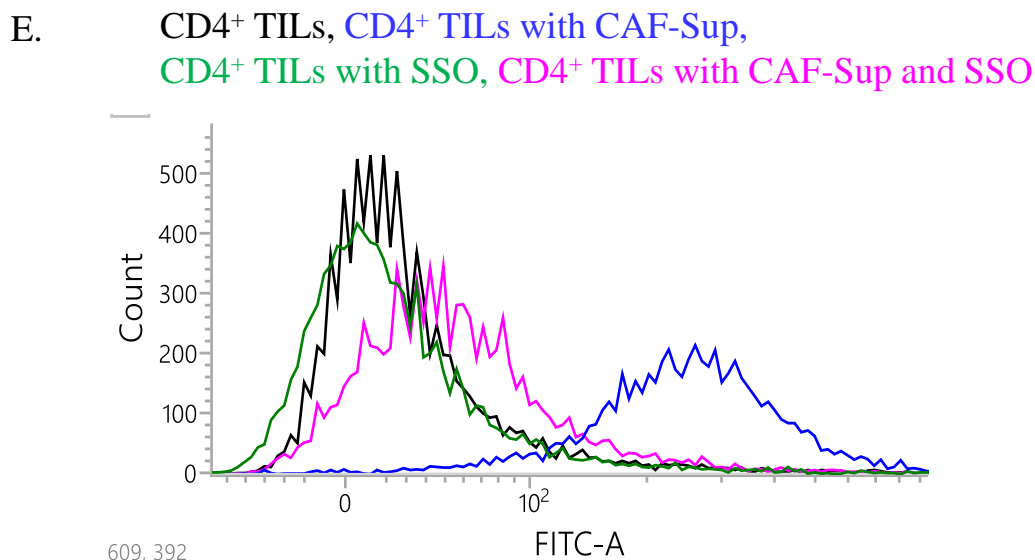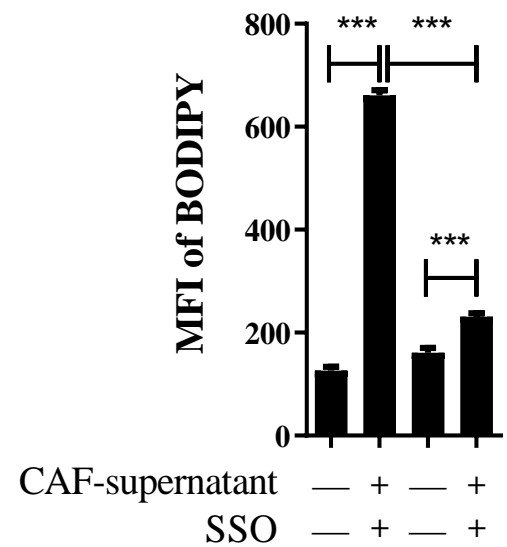

A.

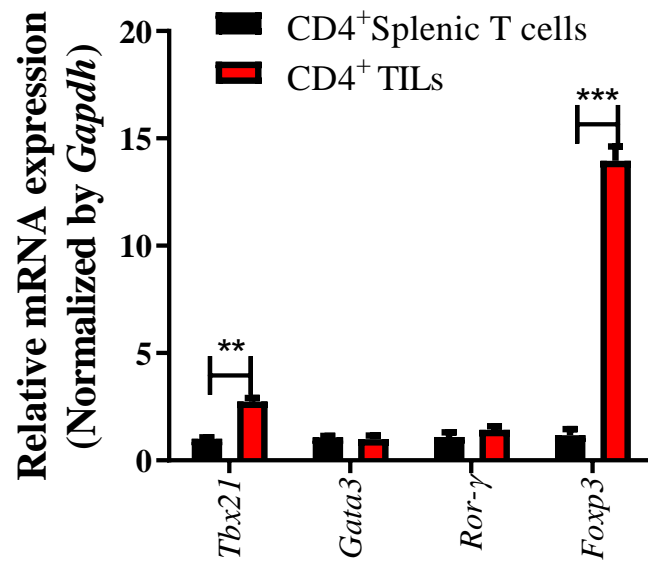

B.

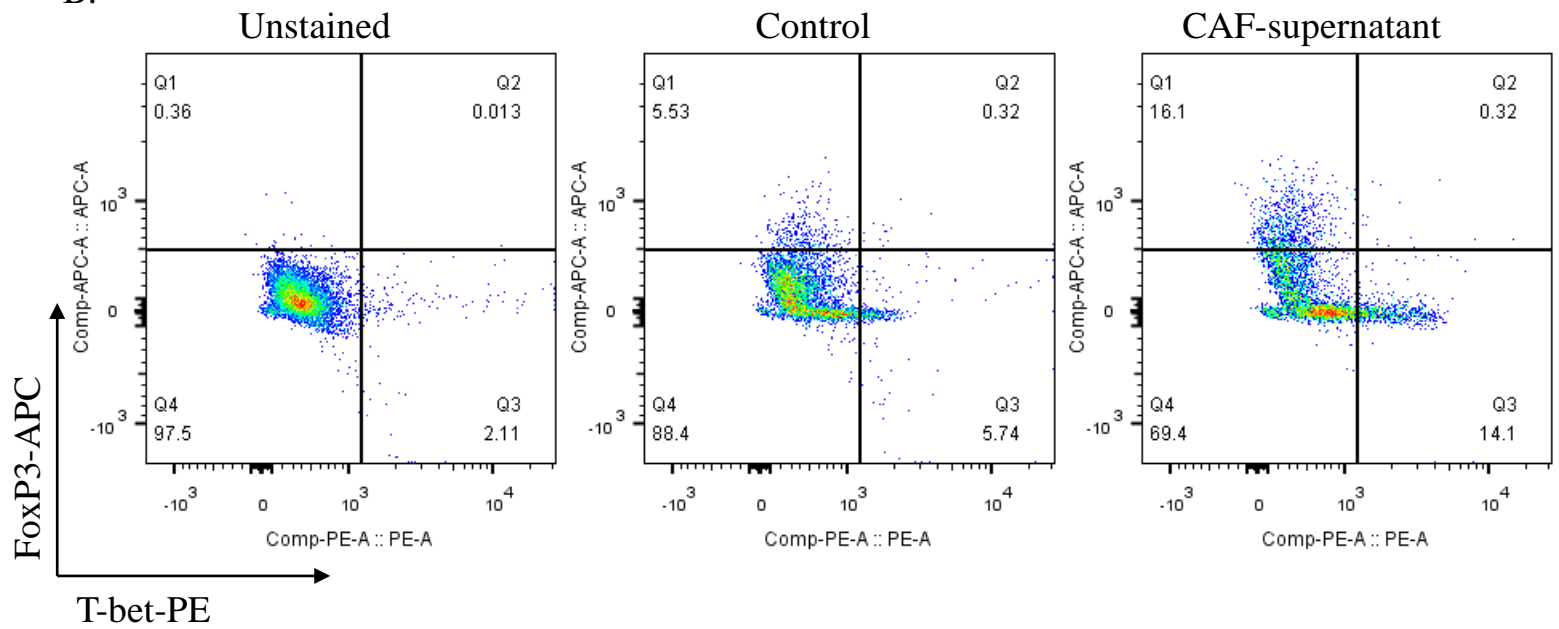

C.

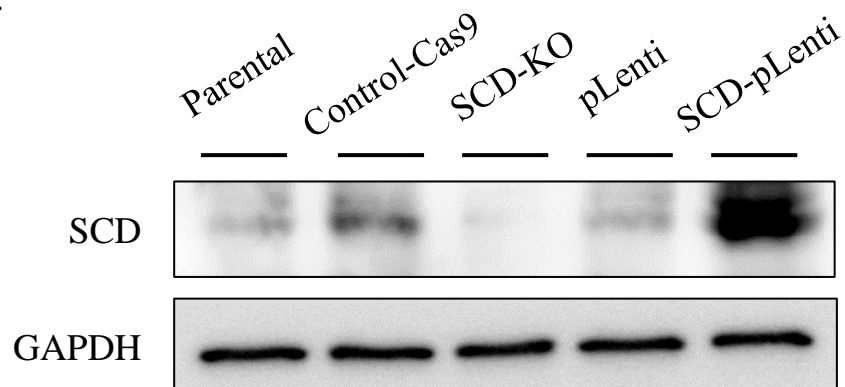

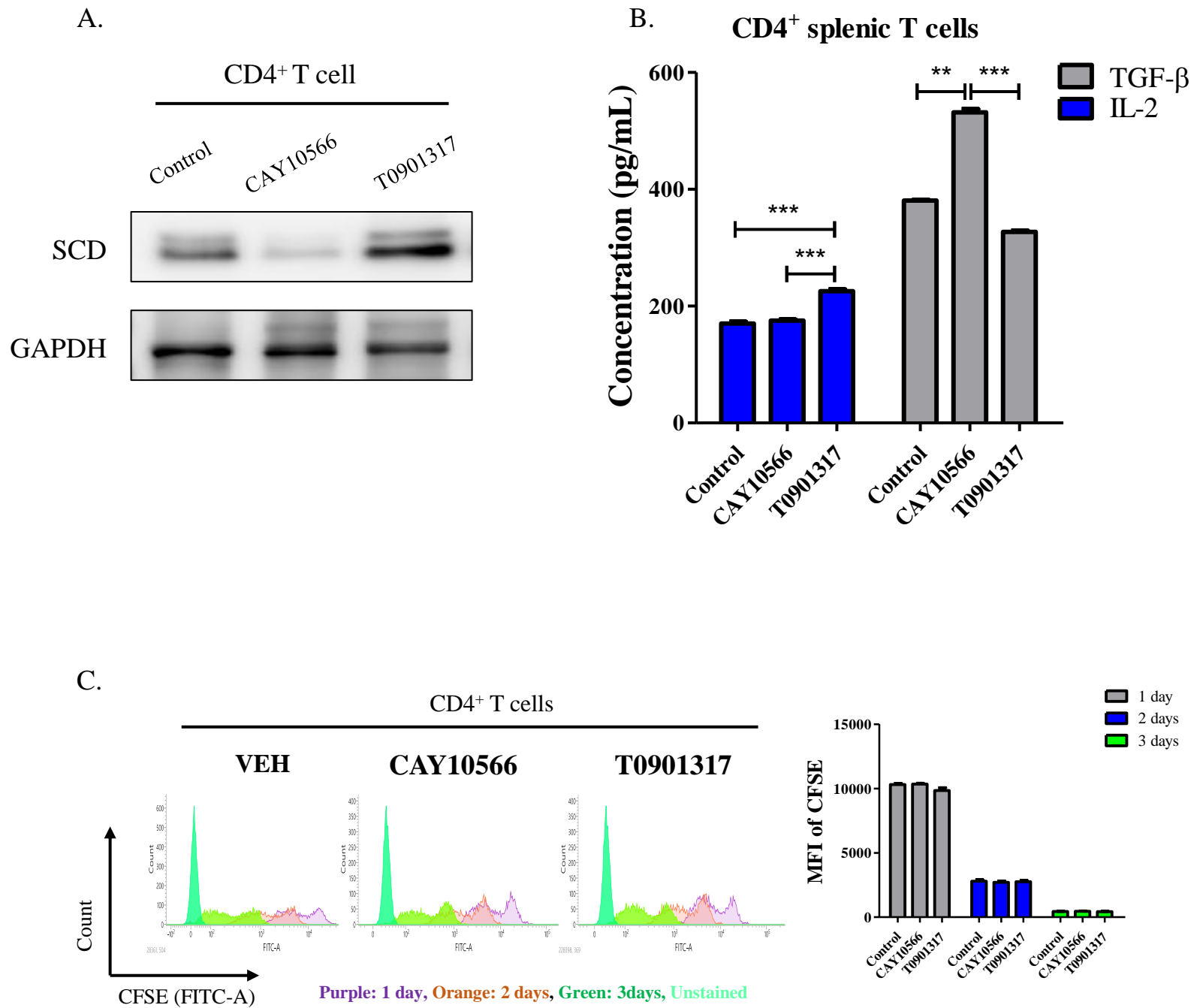

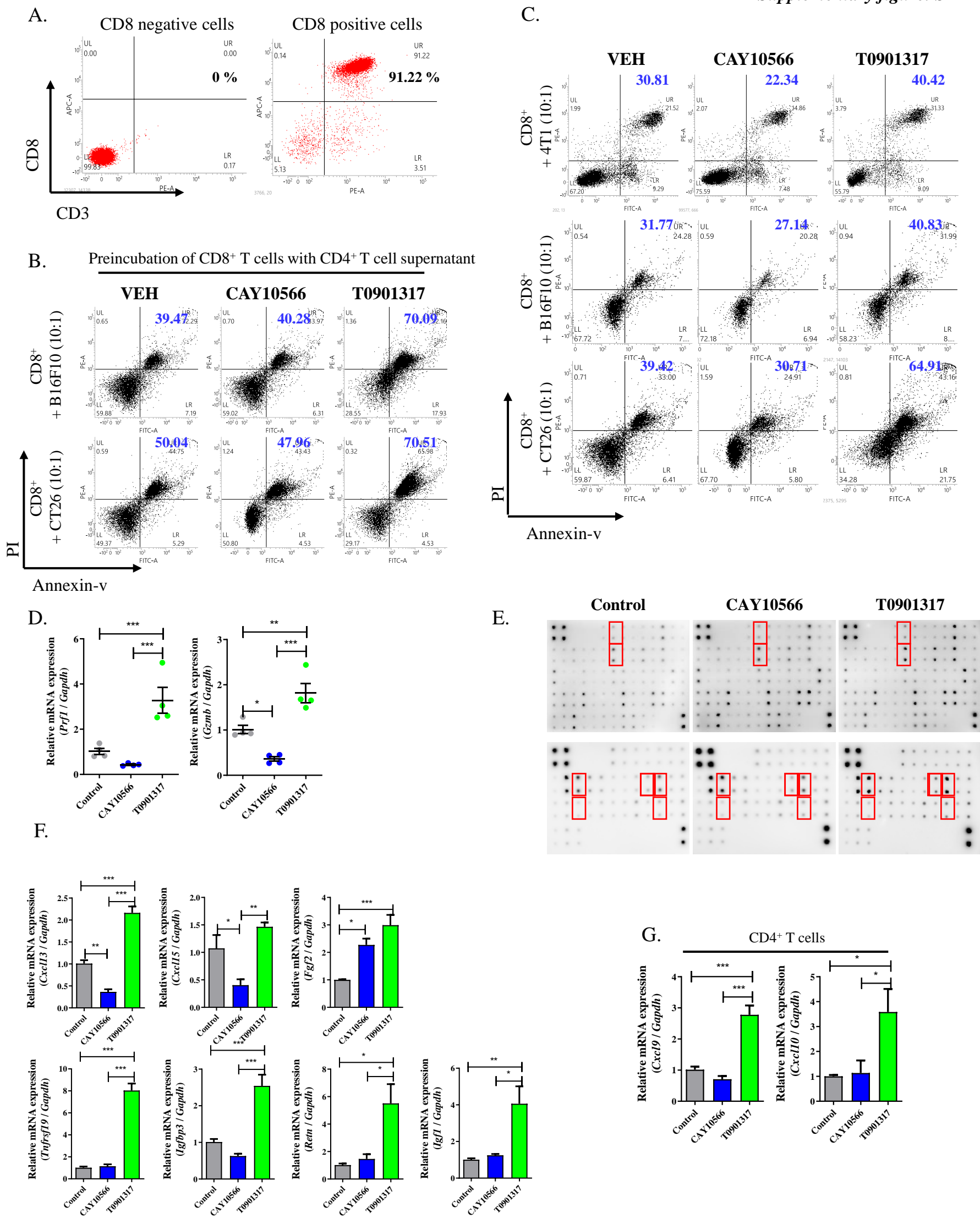

A.

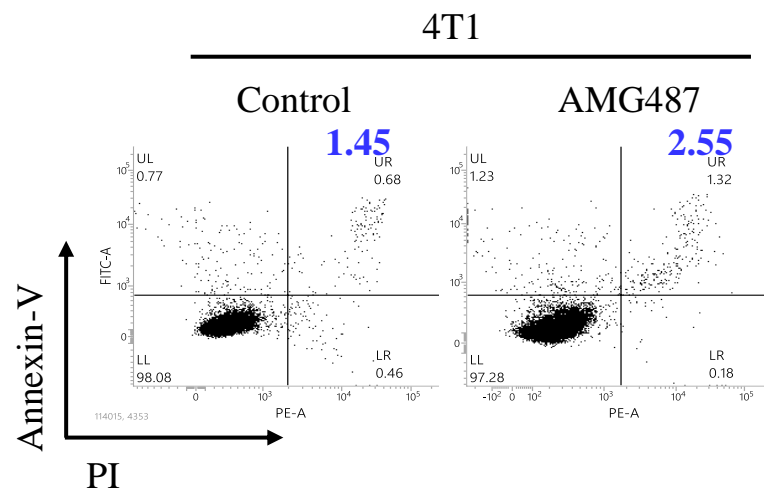

B.

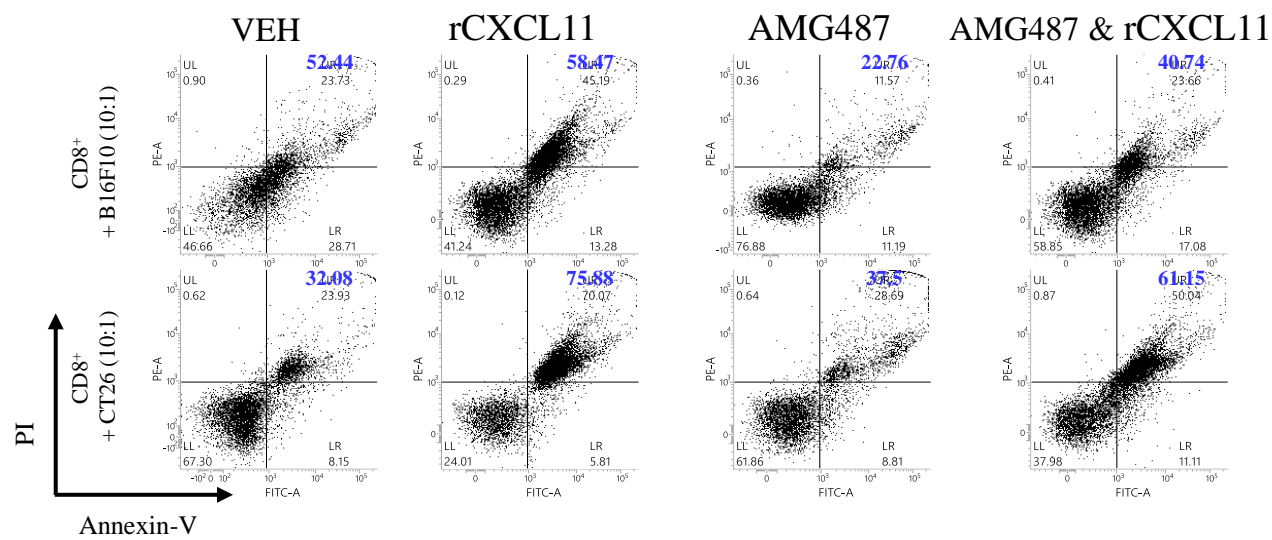

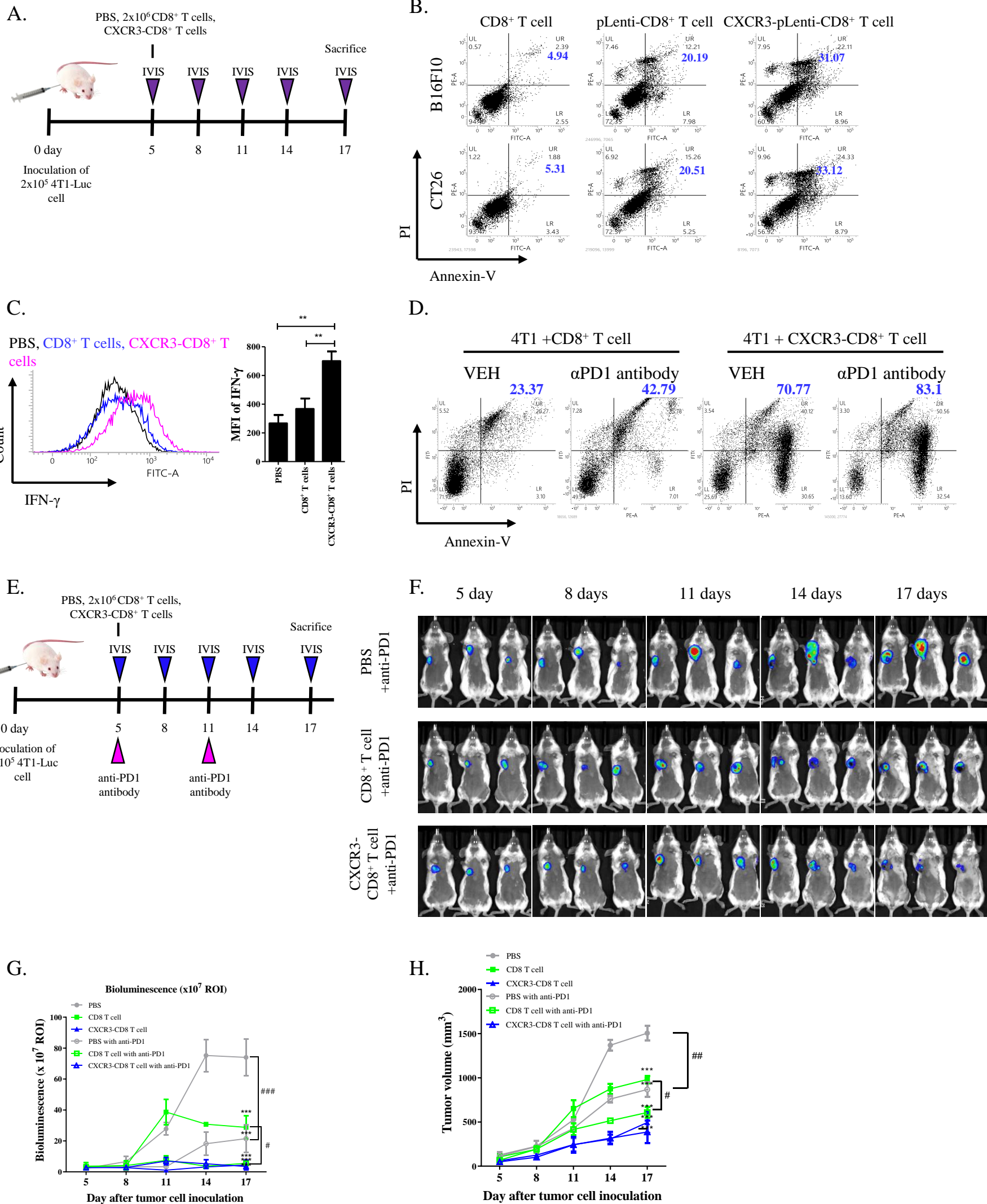

A.

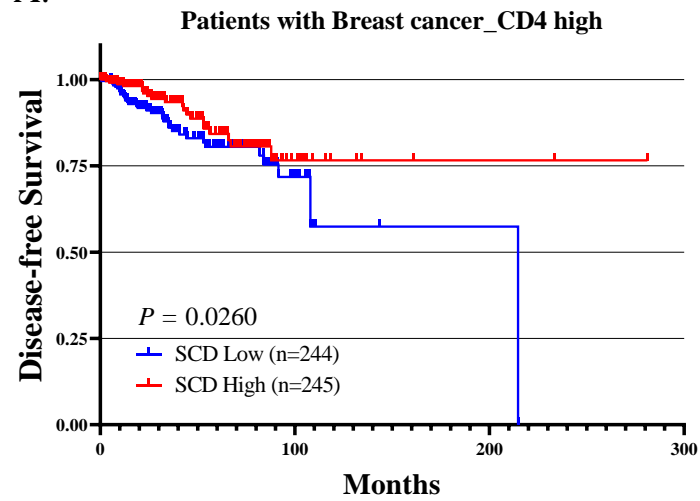

B.

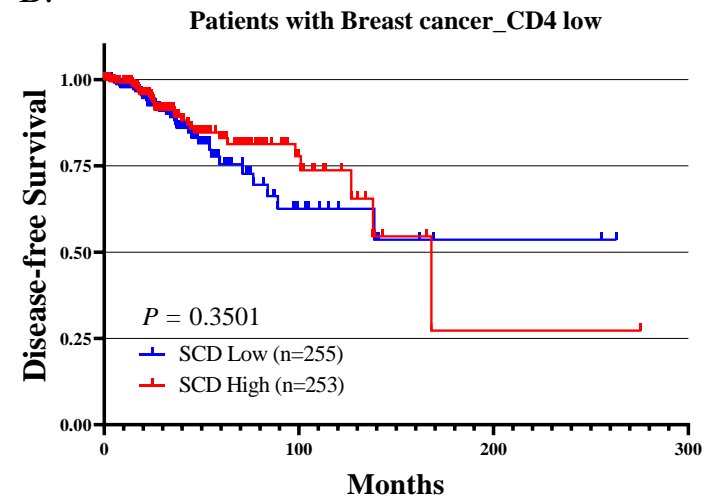

C.

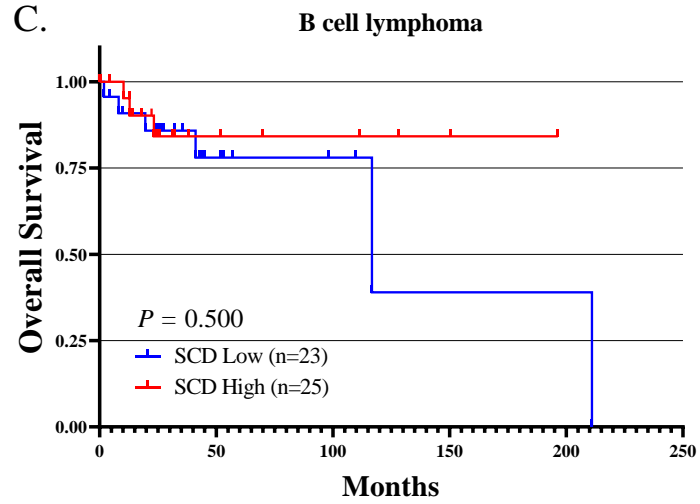

D.

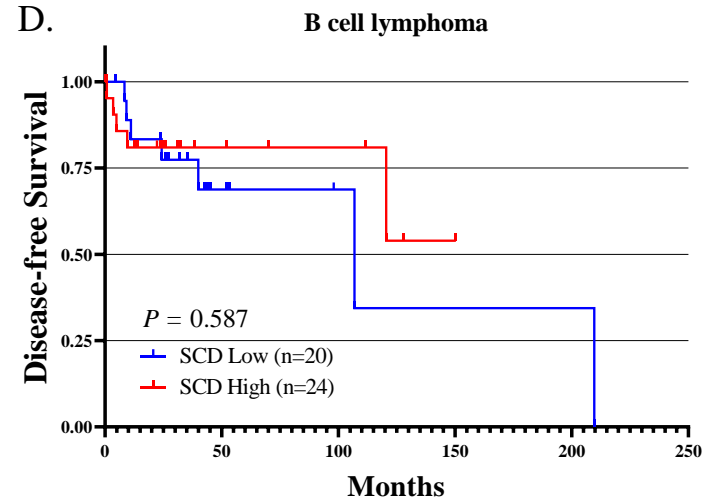

Supplement: Supplementary file 1 — Supplementary figure S1. Characterization of lipid metabolism in TILs incubated with CAFs-supernatant. (A) Representative dot plot image of CD3 and CD4 staining in isolated CD4+ splenocytes and TILs in tumor tissue isolated from 4T1 mouse model. (B) Representative fluorescence image of staining for CD4 and FAP in spleen from normal mouse. Red: CD4, Green: FAP, Blue: DAPI. Scale bar: 100 μm. (C) Representative histogram of FAP staining in isolated CAFs from tumor mass in 4T1 cells. The P4 population was established using negatively isolated cells. ***P<0.001. Data indicate the mean ± SEM (n = 3). (D) Transcription levels of Cd36 and Slc27a1 in CD4+ splenic T cells and TILs incubated with CAF-supernatant for 1 day. *P < 0.05 and ***P < 0.001. Data indicate the mean ± SEM (n = 4). (E) Representative histogram of BODIPY staining in CD4+ TILs treated with CAF-supernatant and SSO. The graph indicated the MFI of BODIPY staining. ***P<0.001. Data indicate the mean ± SEM (n = 3). Supplementary figure S2. SCD regulates the expressions of marker for Treg and Th1 cells and mitochondrial function. (A) Transcription levels of Tbx21, Gata1, Rorγ, and Foxp3 in CD4+ splenic T cells and TILs. **P<0.01 and **P<0.01. Data indicate the mean ± SEM (n = 4). (B) Analysis of FoxP3 and T-bet staining in Jurkat T cells after treatment with CAF-supernatant for 1 day. (C) Expressions of SCD and GAPDH in parental, Control-Cas9-transfected cells, SCD-KO cells, empty-pLenti-infected cells and SCD-pLenti cells. Supplementary figure S3. The effect of SCD in CD4+ splenic T cells. (A) Expression of SCD in CD4+ splenic T cells treated with control, 1 μM CAY10566 and 10 μM T0901317 for 1 day. (B) The concentration of TGF-β and IL-2 in CD4+ splenic T cells treated with control, 1 μM CAY10566 and 10 μM T0901317. **P < 0.01 and ***P < 0.001. Data indicate the mean ± SEM (n = 4). (C) CFSE staining in CD4+ T cells treated with control, 1 μM CAY10566 and 10 μM T0901317 for 1 day. The fluorescent of CFSE stain [file 13578_2024_1308_MOESM1_ESM.pdf]
