# Supplementary material for: Stearoyl-CoA desaturase in CD4+ T cells suppresses tumor growth through activation of the CXCR3/CXCL11 axis in CD8+ T cells
Source: Cell Biosci. 2024 Nov 14;14:137. doi: 10.1186/s13578-024-01308-3 (PMC11566202; doi:10.1186/s13578-024-01308-3)
Supplement: Supplementary file 2 — Supplementary Material 2. [file 13578_2024_1308_MOESM2_ESM.docx]

| *TBX21* | Forward | 5’-ATTGCCGTGACTGCCTACCAGA-3’ |  |  |
| --- | --- | --- | --- | --- |
|  | Reverse | 5’-GGAATTGACAGTTGGGTCCAGG-3’ |  |  |
| *FOXP3* | Forward | 5’-GGCACAATGTCTCCTCCAGAGA-3’ |  |  |
|  | Reverse | 5’-CAGATGAAGCCTTGGTCAGTGC-3’ |  |  |
| *CXCL9* | Forward | 5’-CTGTTCCTGCATCAGCACCAAC-3’ |  |  |
|  | Reverse | 5’-TGAACTCCATTCTTCAGTGTAGCA-3’ |  |  |
| *CXCL10* | Forward | 5’-GGTGAGAAGAGATGTCTGAATCC-3’ |  |  |
|  | Reverse | 5’-GTCCATCCTTGGAAGCACTGCA-3’ |  |  |
| *CXCL11* | Forward | 5’-AAGGACAACGATGCCTAAATCCC-3’ |  |  |
|  | Reverse | 5’-CAGATGCCCTTTTCCAGGACTTC-3’ |  |  |
| *GAPDH* | Forward | 5’-GTCTCCTCTGACTTCAACAGCG-3’ |  |  |
|  | Reverse | 5’-ACCACCCTGTTGCTGTAGCCAA-3’ |  |  |
| *Cd36* | Forward | 5’-GAGCCATCTTTGAGCCTTCA-3’ |  |  |
|  | Reverse | 5’-TCAGATCCGAACACAGCGTA-3’ |  |  |
| *Slc27a1* | Forward | 5’-TGCCACAGATCGGCGAGTTCTA-3’ |  |  |
|  | Reverse | 5’-AGTGGCTCCATCGTGTCCTCAT-3’ |  |  |
| *Tbx21* | Forward | 5’-CCACCTGTTGTGGTCCAAGTTC-3’ |  |  |
|  | Reverse | 5’-CCACAAACATCCTGTAATGGCTTG-3’ |  |  |
| *Gata3* | Forward | 5’-CCTCTGGAGGAGGAACGCTAAT-3’ |  |  |
|  | Reverse | 5’-GTTTCGGGTCTGGATGCCTTCT-3’ |  |  |
| *Rorc* | Forward | 5’-GTGGAGTTTGCCAAGCGGCTTT-3’ |  |  |
|  | Reverse | 5’-CCTGCACATTCTGACTAGGACG-3’ |  |  |
| *Foxp3* | Forward | 5’-CCTGGTTGTGAGAAGGTCTTCG-3’ |  |  |
|  | Reverse | 5’-TGCTCCAGAGACTGCACCACTT-3’ |  |  |
| *Cxcl9* | Forward | 5’-CCTAGTGATAAGGAATGCACGATG-3’ |  |  |
|  | Reverse | 5’-CTAGGCAGGTTTGATCTCCGTTC-3’ |  |  |
| *Cxcl10* | Forward | 5’-ATCATCCCTGCGAGCCTATCCT-3’ |  |  |
|  | Reverse | 5’-GACCTTTTTTGGCTAAACGCTTTC-3’ |  |  |
| *Cxcl11* | Forward | 5’-CCGAGTAACGGCTGCGACAAAG-3’ |  |  |
|  | Reverse | 5’-CCTGCATTATGAGGCGAGCTTG-3’ |  |  |
| *Pfr1* | Forward | 5’-ACACAGTAGAGTGTCGCATGTAC-3’ |  |  |
|  | Reverse | 5’-GTGGAGCTGTTAAAGTTGCGGG-3’ |  |  |
| *Gzmb* | Forward | 5’-CAGGAGAAGACCCAGCAAGTCA-3’ |  |  |
|  | Reverse | 5’-CTCACAGCTCTAGTCCTCTTGG-3’ |  |  |
| *Cxcl13* | Forward | 5’-CATAGATCGGATTCAAGTTACGCC-3’ |  |  |
|  | Reverse | 5’-GTAACCATTTGGCACGAGGATTC-3’ |  |  |
| *Cxcl15* | Forward | 5’-GGTGATATTCGAGACCATTTACTG-3’ |  |  |
|  | Reverse | 5’-GCCAACAGTAGCCTTCACCCAT-3’ |  |  |
| *Fgf2* | Forward | 5’-AAGCGGCTCTACTGCAAGAACG-’ |  |  |
|  | Reverse | 5’-CCTTGATAGACACAACTCCTCTC-3’ |  |  |
| *TNFrsf19* | Forward | 5’-TGTGTCCTCTGCAAACAGTGCG-3’ |  |  |
|  | Reverse | 5’-CCAGTCTTCCTTGAACCGTTGC-3’ |  |  |
| *Igfbp3* | Forward | 5’-CCTCAATGTGCTGAGTCCCAGA-3’ |  |  |
|  | Reverse | 5’-CTTGTCCACACACCAGCAGAAG-3’ |  |  |
| *Retn* | Forward | 5’-CATGCCACTGTGTCCCATCGAT-3’ |  |  |
|  | Reverse | 5’-ACTTCCCTCTGGAGGAGACTGT-3’ |  |  |
| *Igf1* | Forward | 5’-GTGGATGCTCTTCAGTTCGTGTG-3’ |  |  |
|  | Reverse | 5’-TCCAGTCTCCTCAGATCACAGC-3’ |  |  |
| *Gapdh* | Forward | 5’-AACTTTGGCATTGTGGAAGG-3’ |  |  |
|  | Reverse | 5’-CACATTGGGGGTAGGAACAC-3’ |  |  |
| SCD | sgRNA | CCTTCCTTATCCTTGTAGG |  |  |

**Supplementary Table S1. primer sequences for RT-PCR and SCD sequence for manipulation of SCD KO by CRISPR/Cas9 system.**
